# Zinc or Multiple Micronutrient Supplementation to Reduce Diarrhea and Respiratory Disease in South African Children: A Randomized Controlled Trial

**DOI:** 10.1371/journal.pone.0000541

**Published:** 2007-06-27

**Authors:** Kany-Kany Angelique Luabeya, Nontobeko Mpontshane, Malanie Mackay, Honorine Ward, Inga Elson, Meera Chhagan, Andrew Tomkins, Jan Van den Broeck, Michael L. Bennish

**Affiliations:** 1 Africa Centre for Health and Population Studies, University of KwaZulu Natal, Somkhele, South Africa; 2 Church of Scotland Hospital, Tugela Ferry, South Africa; 3 Department of Medical Microbiology, Nelson R. Mandela School of Medicine, University of KwaZulu-Natal, Durban, South Africa; 4 Division of Geographic Medicine and Infectious Diseases, Tufts-New England Medical Centre, Boston, Massachusetts, United States of America; 5 Department of Chemical Pathology, University of KwaZulu-Natal, Durban, South Africa; 6 Department of Paediatrics and Child Health, University of KwaZulu-Natal, Durban, South Africa; 7 Friedman School of Nutrition Science and Policy, Tufts University, Boston, Massachusetts, United States of America; 8 Centre for International Child Health, University of London, London, United Kingdom; 9 Tropical Medicine Research Institute, University of the West Indies, Kingston, Jamaica; Nuffield Department of Clinical Medicine, University of Oxford, Oxford, United Kingdom; Institute of Clinical Effectiveness and Health Policy, Argentina

## Abstract

**Background:**

Prophylactic zinc supplementation has been shown to reduce diarrhea and respiratory illness in children in many developing countries, but its efficacy in children in Africa is uncertain.

**Objective:**

To determine if zinc, or zinc plus multiple micronutrients, reduces diarrhea and respiratory disease prevalence.

**Design:**

Randomized, double-blind, controlled trial.

**Setting:**

Rural community in South Africa.

**Participants:**

Three cohorts: 32 HIV-infected children; 154 HIV-uninfected children born to HIV-infected mothers; and 187 HIV-uninfected children born to HIV-uninfected mothers.

**Interventions:**

Children received either 1250 IU of vitamin A; vitamin A and 10 mg of zinc; or vitamin A, zinc, vitamins B1, B2, B6, B12, C, D, E, and K and copper, iodine, iron, and niacin starting at 6 months and continuing to 24 months of age. Homes were visited weekly.

**Outcome Measures:**

Primary outcome was percentage of days of diarrhea per child by study arm within each of the three cohorts. Secondary outcomes were prevalence of upper respiratory symptoms and percentage of children who ever had pneumonia by maternal report, or confirmed by the field worker.

**Results:**

Among HIV-uninfected children born to HIV-infected mothers, median percentage of days with diarrhea was 2.3% for 49 children allocated to vitamin A; 2.5% in 47 children allocated to receive vitamin A and zinc; and 2.2% for 46 children allocated to multiple micronutrients (P = 0.852). Among HIV-uninfected children born to HIV-uninfected mothers, median percentage of days of diarrhea was 2.4% in 56 children in the vitamin A group; 1.8% in 57 children in the vitamin A and zinc group; and 2.7% in 52 children in the multiple micronutrient group (P = 0.857). Only 32 HIV-infected children were enrolled, and there were no differences between treatment arms in the prevalence of diarrhea. The prevalence of upper respiratory symptoms or incidence of pneumonia did not differ by treatment arms in any of the cohorts.

**Conclusion:**

When compared with vitamin A alone, supplementation with zinc, or with zinc and multiple micronutrients, did not reduce diarrhea and respiratory morbidity in rural South African children.

**Trial Registration:**

ClinicalTrials.gov NCT00156832

## Introduction

Micronutrient deficiencies are common in children in developing countries, including children in South Africa.[1]–[3] Zinc deficiency is especially thought to be associated with an increased susceptibility to infection, and a greater severity of disease when infection occurs.[4] Zinc is now recommended by the WHO for the treatment of children with diarrhea in developing countries,[5] but its role for the prophylaxis of infection is less certain.

Studies in Asia and Latin American have generally found that prophylactic zinc reduces the incidence, duration and severity of diarrhea and pneumonia episodes when given to infants and young children.[6] Few studies have been conducted of prophylactic administration of zinc in Africa,[7], [8] which differs, especially from Asia, in diet and patterns of growth in children,[9] and in the burden of infectious diseases, especially HIV. Because micronutrient deficiencies are seldom isolated to a single micronutrient, there have also been efforts to provide supplements containing an array of micronutrients.[1]

In KwaZulu-Natal, where this study was conducted, antenatal HIV prevalence is 39%.[10] Children with HIV infection are known to be at high risk of infection and poor nutrition and growth,[11] and those born to HIV-infected mothers but not themselves infected may be at increased risk of nutritional deficiencies because of parental illness.

## Methods

The protocol for this trial and supporting CONSORT checklist are available as supporting information; see Checklist S1 and Protocol S1.

### Study area

The study was conducted in northern KwaZulu-Natal Province, South Africa. Median household income in the area is one-half the national average, 80% of the population lives below the national poverty line, and unemployment is 75%.[12] There is little subsistence farming in this area despite 98% of households being defined as rural, and food insecurity within households is common.[12] Most infants receive both breast and complementary feeding from early infancy,[13] and the diet is known to be deficient in micronutrients.[14]

### Participants

Children were enrolled into the study by nurses at five government primary health care clinics. Study subjects were recruited from children coming to the clinics for routine visits, from households identified as having potentially eligible children based on surveillance conducted by the Africa Centre Demographic Information System, and from children enrolled at birth in an observational study of the determinants of mother-to-child transmission of HIV.

Children eligible for study were four to six months old. Children were excluded from study if they were: less than 60% of median weight-for-age using United States National Center for Health Statistics standards;[15] had nutritional edema; had received vitamin or micronutrient supplements in the previous month; had diarrhea for more than seven days at the time of study enrollment; or were enrolled in another study of a clinical intervention.

To maintain community blinding to the HIV status of women enrolled in the study, we enrolled children in any cohort which reached targeted enrollment before enrollment was completed in other cohorts, provided them only their initial two weeks of supplement, and did not include them in the analysis.

### Ethics and study monitoring

The study was approved by the Ethics Review Committee of the University of KwaZulu-Natal and the Institutional Review Board of Tufts-New England Medical Center. Before initiation the study was presented to the Community Advisory Board of the Africa Centre for their review and advice, and interim reports on study progress were provided to this Advisory Board. The study was also monitored by a Data Monitoring and Safety Board established by the NIH. Written informed consent to participate in the study, and to do HIV testing on both the mother and child, was obtained from all mothers whose children participated in the study.

### Intervention

The three treatment arms were: vitamin A alone; vitamin A plus zinc; and vitamin A, zinc and multiple micronutrients. All supplements were given daily at home from entry into the study until 24 months of age. Vitamin A was given as the comparator regimen, rather than placebo, because the South African Department of Health guidelines recommend routine three-monthly vitamin A supplementation for children and clinics did not consistently provide vitamin A to children. Thus each of the three tablet formulations contained 1250 IU of vitamin A. Two of the three tablet formulations contained 10 mg of zinc as zinc gluconate. The multiple micronutrient tablets were similar to those used in recent international trials of multiple micronutrient supplementation,[16] and in addition to vitamin A and zinc contained : 0.5 mg each of vitamins B1, B2 and B6; 0.9 µg vitamin B12; 35 mg vitamin C; 5 µg vitamin D; 6 mg vitamin E; 10 µg vitamin K; 0.6 mg copper as cupric gluconate; 150 µg folate; 50 µg iodine; 10 mg iron as ferrous fumerate; and 6 mg niacin. The supplements were manufactured as tablets by Hersil Ltd. (Lima, Peru) and packaged in blister packs of seven tablets. All three formulations were similar in color, taste, appearance and size. Because of a delay in shipment, 243 children enrolled in the study did not receive supplements for 11 weeks in 2005.

### Objectives

To study objective was to determine if zinc, or zinc plus multiple micronutrients, would reduce diarrhea and respiratory disease prevalence. We hypothesized that both zinc, and zinc plus multiple micronutrients, would reduce diarrhea and respiratory disease prevalence and examined their efficacy in a randomized controlled trial in children 6–24 months of age born to both HIV-infected and HIV-uninfected mothers.

### Study Outcomes

The primary study outcome was the percentage of days of diarrhea per child compared between study arms within each of the three cohorts. Secondary diarrheal disease outcomes were measures of the severity of diarrhea (duration of episodes, maximum number of stools during an episode, episodes lasting 14 or more days, diarrhea episodes with blood in the stool, and clinic visits for diarrhea) and the distribution of diarrheal morbidity (proportion of children who ever had diarrhea and number of episodes per child). Respiratory disease outcome measures were the percentage of weeks of upper respiratory symptoms per child, the percentage of children who ever had pneumonia by maternal report, and the percentage of children with pneumonia confirmed by the field worker.

### Study definitions

Diarrhea was considered to be present on any day the stool was more frequent or looser than normal or the stool contained water or blood. An episode of diarrhea terminated on the last day of diarrhea that was followed by two consecutive days without diarrhea. Blood was considered present in stool if reported by the mother. Upper respiratory tract infection was defined as the history (or presence during the field worker visit) of cough or runny nose. Because field workers asked about the occurrence of these signs since the last visit, rather than by day, the outcome was defined as prevalent weeks. Pneumonia by maternal report was considered to have occurred if there was a history of either fast breathing or chest in-drawing. Confirmed pneumonia was defined as an elevated respiratory rate at rest measured by the fieldworker using WHO/UNICEF Integrated Management of Childhood Illness guidelines.[5]

### Sample size

This was done separately for each of the three cohorts and was based on percentage of days of diarrhea for each child by cohort and treatment arm. We assumed the percentage of days with diarrhea in HIV-infected children receiving vitamin A alone would be 4.4% (SD±0.9%); for those receiving vitamin A and zinc it would be 3.7%±0.9% (17% less than for those receiving vitamin A alone); and for those receiving multiple micronutrients it would be 3.0%±0.9% (33% less than for those receiving vitamin A alone and 20% less than for those receiving vitamin A and zinc). For HIV-uninfected children we postulated the same differences between treatment groups but with lower diarrhea prevalence rates: 3.3%±0.9% (vitamin A alone); 2.8%±0.9% (vitamin A and zinc); and 2.2%±0.9% (multiple micronutrient group). To determine with a power of 0.8 and a two-sided significance level of .05 if these postulated differences existed, 26 children were required in each of the three arms of the HIV-infected cohort, and 45 children in each of the three arms of the two cohorts of HIV-uninfected children. We expected mortality, migration, and withdrawals to diminish days of observation by 50% in the HIV-infected cohort and 25% in the HIV-uninfected groups. Thus the total sample size required was 156 in the HIV-infected cohort and 180 children in each of the two cohorts of HIV uninfected children.

### Randomization–sequence generation

An allocation list was prepared using computer-generated random numbers and a block size of six. Assignment to the three treatment arms was done separately for three cohorts of children stratified by HIV status of child and mother: HIV-infected children and mothers; HIV-uninfected children of HIV-infected mothers; and HIV-uninfected children of HIV-uninfected mothers.

### Randomization–allocation concealment and implementation

The manufacturer prepared numbered packs of tablets corresponding to the allocation list. Children enrolled in the study were assigned by a study physician to one of the three study cohorts after results of the HIV tests became available. The physician then allocated the next pack of tablets from the blocks assigned to that cohort to the participant.

### Blinding

Investigators, study staff and participants were blind to the treatment assignments.

### Study procedures

At enrollment study subjects had a physical examination and medical history taken by the study nurse. Weight was measured to a precision of 0.1 kg using an electronic digital scale and recumbent length to a precision of 0.1 cm using an infantometer. For determination of anthropometric centiles National Center for Health Statistics standards were used.[15] Mothers and children had a blood sample taken for determination of HIV infection status if their status was not already known and documented, and for determining haemoglobin concentration. A two-week supply of tablets were provided at enrolment, and the mothers instructed on administering tablets to their child by crushing it and mixing it with food, and assuring that the child ate all the food the supplement was mixed in.

Trained fieldworkers visited the children at home weekly during the study. They supplied sufficient tablets to ensure a two week supply was on hand. Any tablets remaining from the previous supply were counted and recorded. At each home visit field workers observed the preparation and administration of a tablet, and waited for 30 minutes to observe any adverse effect.

At each visit field workers administered a standardized questionnaire that focused on diarrheal and respiratory morbidity using locally validated terms.[17] If the mother reported diarrhea then the field worker asked for each day since the last visit if the stool was looser or more frequent than normal, contained water or blood, and the number of stools on the worst day of diarrhea. Mothers were asked if since the last visit the child had a runny nose or cough, difficult or fast breathing, or chest in-drawing. Field workers measured the respiratory rate at rest in any child with current or reported signs or symptoms of respiratory distress.

If the mother or another informant familiar with the child was not available during the scheduled home visit two further attempts were made to visit that week, following which the next planned weekly visit was made. Field workers collected information on events that occurred a maximum of 28 days before the visit.

Mothers and infants were requested to return for clinic visits at 7, 8, 9, 12, 15, 18, 21 and 24 months of age to assess growth and morbidity. Ill children were referred to the study nurses at the clinics, or for more serious illness to the district hospital. When antiretroviral therapy for children became available in clinics in January 2005 all children with HIV infection in the study were referred for evaluation.

Supervisors assessed reliability of field worker assessments by conducting unscheduled visits and comparing findings with interviews conducted the same day by field workers

### Laboratory methods

HIV testing of children was done using a quantitative HIV RNA assay (Nuclisens HIV-1 QT, Organon Teknika or Nuclisens EasyQ HIV-1, Biomerieux, Boxtel, The Netherlands). Maternal HIV status was determined using two ELISA tests (first Vironostika HIV-1 Microelisa system and then Uni-Form II plus O if the first test was positive, both Biomèrieux) each of which had to be positive for HIV infection to be diagnosed. Hemoglobin determination was done at the clinic by study nurses using a portable HemoCue system (Angelholm, Sweden).

### Statistical methods

Data were entered into an electronic data base using an optical form scanning system (TeleForm version 7.1 Cardiff, Vista CA, USA) and were analyzed using SPSS version 13.0 (SPSS Inc., Chicago, IL, USA) and EpiInfo version 3.3.2 (Centers for Disease Control and Prevention, Atlanta, GA, USA).

Analyses were done on a locked dataset using an analysis plan, and tests of significance, that were determined before the analyses were conducted. Analyses were initially conducted using coded treatment groups with the analysts blinded to the actual treatment.

Analysis of all outcomes was on an intention-to-treat basis–all participants for whom there were observations on study outcomes were included in the analysis. In addition to comparing baseline characteristics between children in the treatment groups, we also examined features during study that could have confounded study outcome. These were rates of drop-out and death, proportion of missing information, dietary pattern, adherence to treatment, and intake of other vitamin and micronutrient preparations. Any baseline or during treatment characteristics that differed significantly between treatment arms was tested for its association with the study outcome using Spearman's rank correlation test.

The primary outcome–percentage of days of diarrhea per child by cohort and treatment group-was not normally distributed as determined by the Kolmogorov-Smirnov test, and a Kruskall-Wallis test was used to determine the overall significance of difference between treatment arms. For secondary outcomes and for baseline characteristics the significance of differences for continuous variables was tested using one-way ANOVA if the variable was normally distributed or the Kruskall-Wallis test if not normally distributed. Differences in proportions were tested with the χ^2^ test. Comparisons between individual groups were made only if the overall group comparison found a significant difference. To determine the effect size for the primary outcome, we determined the difference between medians, and the 95% confidence intervals for those differences, using the binomial method.[18] All tests of differences or association were two-sided, and were considered significant if the P value was ≤ 0.05.

We also tested for any bias in assignment to treatment cohort (on the assumption that blinding may not have been completely effective) using the Berger-Exner test.[19]

## Results

### Recruitment, patient flow, and numbers analyzed

**Figure 1:**
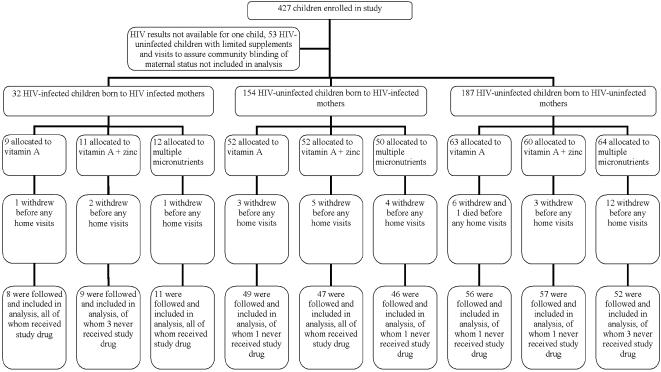
Patient Flowchart

Study enrollment began in June 2003 and the last participant enrolled in October 2004. Patient observations were completed 31 January 2006. Three-hundred seventy-three children were enrolled in the study–32 HIV-infected children, 154 children without HIV infection born to HIV-infected mothers, and 187 children born to HIV-uninfected mothers (Figure 1). Thirty-seven children withdrew and one died before any home visits took place, leaving 335 children for whom information on outcomes was available and who were included in the analysis (Figure 1). One-hundred thirteen of these 335 children each received vitamin A alone or vitamin A plus zinc, and 109 of the children received multiple micronutrients. Ten of these 335 children never took the supplement but were included in this intention-to-treat analysis. Berger-Exner tests performed in each cohort separately showed no evidence of bias in assignment to treatment group. Controlling for treatment arm, the P-values for the partial correlation between percent days of diarrhea (the main outcome) and the remaining proportion of multivitamin tablets in the block at the time of random assignment to therapy was >0.23 in each of the three cohorts.

Twelve (3.6%) of the 335 children with at least one home visit died during the study, including 8 (29%) of the 28 children with HIV infection. An additional 88 (26.2%) of the 335 children who had at least one home visit did not complete the study (Table 1). Fifty-seven children moved out of the area during the study. Reasons given for withdrawal in the other 31 children included lack of time by parent to participate (2 children), the child not liking the taste of the tablets (3 children), objections from grandparent or father (2 children) and unspecified reasons in 24 children. The median duration of enrollment in the study was 447 days and did not differ significantly between groups.

**Table 1:** Characteristics of 335 children by study cohort and treatment arm

Characteristic	HIV-infected children				HIV-uninfected children born to HIV-infected mothers				HIV-uninfected children born to HIV-uninfected mothers			
	Treatment group			P	Treatment group			P	Treatment group			P
	Vitamin A (n = 8)	Vitamin A+zinc (n = 9)	Multiple micronutrients (n = 11)		Vitamin A (n = 49)	Vitamin A+zinc (n = 47)	Multiple micronutrients (n = 46)		Vitamin A (n = 56)	Vitamin A+zinc (n = 57)	Multiple micronutrients (n = 52)	
Age at enrollment into study, months	5.5 (0.4)	5.4 (0.5)	5.5 (0.3)	0.993	5.4 (0.5)	5.5 (0.6)	5.6 (0.5)	0.372	5.5 (0.5)	5.5 (0.5)	5.4 (0.5)	0.535
Age at initiation of therapy, months	8.0 (1.8)	7.4 (1.0)	8.0 (2.2)	0.737	7.8 (1.3)	8.0 (1.3)	7.8 (1.3)	0.760	7.6 (1.5)	7.3 (0.8)	7.2 (0.9)	0.174
Male, n (%)	6 (75.0)	5 (55.6)	4 (36.4)	0.247	24 (49.0)	25 (53.2)	21 (45.7)	0.767	35 (62.5)	25 (43.9)	28 (53.8)	0.139
Piped or well water used for preparing feeds, n (%)	6 (85.7)	5 (83.3)	6 (75.0)	0.857	36 (76.6)	36 (80.0)	33 (71.7)	0.649	43 (82.7)	40 (70.2)	44 (88.0)	0.059
Anthropometry												
Weight for age Z-score‡	−0.02 (1.83)	−1.05 (1.35)	−0.12 (1.61)	0.340	0.21 (1.24)	0.28 (1.23)	0.08 (1.18)	0.722	0.34 (1.21)	0.54 (1.00)	0.70 (1.17)	0.260
Length for age Z-score‡	−1.37 (0.88)	−1.00 (1.42)	−1.00 (1.19)	0.791	−0.64 (0.82)	−0.50 (0.98)	−0.74 (1.09)	0.514	−0.36 (0.87)	−0.34 (1.02)	−0.37 (0.89)	0.990
Weight for length Z-score‡	0.80 (2.08)	−0.03 (0.74)	0.51 (1.36)	0.535	0.87 (1.29)	0.86 (0.94)	0.74 (1.01)	0.842	0.61 (1.06)	1.00 (0.95)	1.10 (1.13)	0.051
Hemoglobin, g/dl§	10.0 (1.4)	9.8 (0.7)	8.8 (2.5)	0.483	10.3 (1.3)	10.5 (1.3)	10.1 (1.1)	0.598	9.9 (1.2)	10.2 (1.1)	10.3 (1.2)	0.363
Withdrew from study or lost to follow-up, n (%)	2 (25.0)	1 (11.1)	3 (27.3)	0.653	10 (20.4)	12 (25.5)	14 (30.4)	0.532	15 (26.8)	14 (24.6)	17 (32.7)	0.624
Days of observation per child, median (25^th^–75^th^ centiles)	306 (107–536)	181 (62–485)	347 (49–527)	0.912	464 (318–510)	418 (255–478)	395 (270–501)	0.165	467 (270–515)	486 (342–530)	439 (309–529)	0.535
Percent of study days for which information not available, median (25^th^–75^th^ centiles) ¶	3.1 (0.3–7.1)	1.3 (0.0–15.9)	2.0 (0.0–14.7)	0.967	4.7 (1.0–14.2)	7.7 (2.2–21.4)	5.5 (1.3–19.7)	0.502	4.2 (0.7–12.2)	4.6 (1.4–10.2)	2.3 (0.4–9.2)	0.651
Percent of study days supplement not given median (25^th^–75^th^ centiles), \\	22.0 (14.6–29.0)	11.6 (2.5–28.1)	33.7 (12.3–100)	0.073	25.8 (17.9–35.2)	25.5 (18.0–33.1)	22.7 (13.8–34.9)	0.421	24.2 (16.3–32.7)	22.8 (14.2–30.3)	21.6 (14.0–31.5)	0.527
Percent of study days for which recall period ≤ 7 days	72.1 (56.5–79.1) P = 0.858	69.6 (56.7–92.2)	76.8 (63.7–80.5)	0.858	76.8 (62.7–84.5)	74.1 (62.5–80.0)	74.9 (67.0–82.3)	0.434	77.3 (70.4–84.7)	76.9 (69.0–81.7)	75.8 (63.6–83.7)	0.553

Values are mean (standard deviation) or n (%) unless noted. P values are for the overall group comparison using one-way ANOVA, Kruskall-Wallis or 2 x 3 χ^2^ test.

* Information on water use was not available in the cohort of HIV-infected children for 1 child in the vitamin A group, 3 children in the vitamin A+zinc group, and 1 child in the multiple micronutrient group. In the cohort of HIV-uninfected children born to HIV-infected mothers, information was missing for 2 children in the vitamin A group and 2 children in the multiple micronutrient group. In the cohort of HIV-uninfected children born to HIV-uninfected mothers, information was missing for 4 children in the vitamin A group, and 2 children in the multiple micronutrient group.

‡ As a Z-score of NCHS standards[15]

§ Hemoglobin values were not available in the cohort of HIV-infected children for 5 children in each of the three treatment groups. In the cohort of HIV-uninfected children born to HIV-infected mothers, hemoglobin determinations were not available for 26 children who received vitamin A, 28 children who received vitamin A+zinc, and 20 children who received multiple micronutrients. In the cohort of HIV-uninfected children born to HIV-uninfected mothers, hemoglobin measurements were not available for 34 children in the vitamin A group, 32 children in the vitamin A+zinc group, and 33 children in the multiple micronutrient group.

¶ Days study drug was not given were determined during weekly home visits.

\\ Excludes days after withdrawal of study subjects for which home visits were not permitted, lost to follow-up, or death.

### Baseline data

Baseline demographic characteristics of the treatment groups in the three cohorts did not significantly differ with the exception of weight-for-length in the group of HIV-uninfected children born to HIV-uninfected mothers (Table 1).

### Outcomes

#### Diarrheal morbidity

There was no significant difference between treatment groups in the primary outcome-percentage of days with diarrhea–in any of the three cohorts (Tables 2 and 3). The median percentage of days with diarrhea ranged from 3.4% to 7.1% in the three treatment arms in HIV-infected children, and from 1.8% to 2.7% in the treatment arms in the two cohorts of HIV-uninfected children. In all comparisons the 95% CI for the differences between groups crossed zero (Table 3). There was no difference between treatment arms in any of the secondary outcome measures of diarrhea severity or frequency (Table 2). Children enrolled in the study had a median of 2 episodes of diarrhea while in the study. During 352 person-years of observation for the children in the study there were a total of 1115 episodes of diarrhea, or 3.2 per year of observation. Only 28 children (8.4%) had episodes of persistent diarrhea lasting >14 days, and 79 (23.6%) ever had blood in their stool.

**Table 2:** Outcomes of 335 children with at least one home visit by study cohort and treatment arm

Characteristic	HIV-infected children				HIV-uninfected children born to HIV-infected mothers				HIV-uninfected children born to HIV-uninfected mothers			
	Treatment Group			P	Treatment Group			P	Treatment Group			P
	Vitamin A (n = 8)	Vitamin A+zinc (n = 9)	Multiple micronutrients (n = 11)		Vitamin A (n = 49)	Vitamin A+zinc (n = 47)	Multiple micronutrients (n = 46)		Vitamin A (n = 56)	Vitamin A+zinc (n = 57)	Multiple micronutrients (n = 52)	
**Primary outcome**												
Percentage of days with diarrhea per child	3.4 (0.8–9.4)	7.1 (2.4–12.3)	5.9 (1.5–16.1)	0.668	2.3 (0.7–4.0)	2.5 (0.8–4.4)	2.2 (0.8–5.5)	0.852	2.4 (0.9–5.0)	1.8 (0.5–5.1)	2.7 (0.5–4.6)	0.857
**Secondary outcomes-diarrhea**												
Children who ever had diarrhea, n (%)	7 (87.5)	8 (88.9)	9 (81.8)	0.891	44 (89.8)	39 (83.0)	40 (87.0)	0.616	47 (83.9)	45 (78.9)	40 (76.9)	0.642
Children who ever had blood in stools, n (%)	2 (25.0)	3 (33.3)	6 (54.5)	0.388	11 (22.4)	12 (25.5)	11 (23.9)	0.939	13 (23.2)	11 (19.3)	10 (19.2)	0.838
Diarrhea episodes per child	2 (1–7)	3 (2–7)	3 (2–5)	0.790	2 (1–5)	2 (1–4)	2 (1–4)	0.956	3 (1–6)	2 (1–4)	3(1–5)	0.719
Duration of diarrhea episode, days	3.0 (2.3–5.6)	4.4 (3.1–5.5)	6.3 (2.8–7.8)	0.260	3.0 (2.5–4.0)	4.0 (2.5–5.3)	3.9 (2.4–5.1)	0.353	3.2 (2.5–5.0)	3.9 (2.8–4.9)	4.0 (2.0–4.7)	0.515
Children with diarrhea episodes ≥14 d, n (%)	0	3 (33.3)	3 (27.3)	0.206	3 (6.1)	3 (6.4)	6 (13.0)	0.395	2 (3.6)	5 (8.8)	3 (5.8)	0.508
Children with >5 stools/day on worst day of diarrhea episode*	3/7 (42.9)	4/8 (50.0)	5/9 (55.6)	0.881	18/44 (40.9)	20/39 (51.3)	15/40 (37.5)	0.436	19/47 (40.4)	19/45 (42.2)	23/40 (57.5)	0.226
Children who visited clinic for diarrhea, n (%)	3 (37.5)	6 (66.7)	9 (81.8)	0.136	28 (57.1)	19 (40.4)	23 (50.0)	0.260	30 (53.6)	23 (40.4)	20 (38.5)	0.220
**Secondary outcomes–respiratory illness**												
Percentage of weeks with upper respiratory symptoms	12.4 (2.0–36.2)	29.0 (10.1–50.0)	23.7 (20.2–33.3)	0.222	13.1 (7.8–24.7)	14.0 (6.2–23.0)	14.3 (7.8–22.4)	0.940	16.6 (9.0–22.9)	15.8 (9.0–24.2)	14.2 (7.8–25.4)	0.766
Children who ever had pneumonia by maternal report, n (%)	3 (37.5)	4 (44.4)	6 (54.5)	0.755	11 (22.4)	14 (29.8)	5 (10.9)	0.079	9 (16.1)	12 (21.1)	10 (19.2)	0.791
Children who ever had pneumonia confirmed by measured respiratory rate, n (%)	3 (37.5)	1 (11.1)	4 (36.4)	0.371	8 (16.3)	9 (19.1)	2 (4.3)	0.084	6 (10.7)	10 (17.5)	5 (9.6)	0.397
**Other secondary outcomes**												
Died during study, N, (%)	2 (25.0)	4 (44.4)	2 (18.2)	0.418	0	1 (2.1)	1 (2.2)	0.586	1 (1.8)	1 (1.8)	0	0.628
Admitted to hospital for any reason during study	0	2 (22.2)	4 (36.4)	0.162	1 (2.0)	4 (8.5)	4 (8.7)	0.312	3 (5.4)	4 (7.0)	3 (5.8)	0.929

Values are median (25^th^–75^th^ centiles) or n (%). P values are for the overall group comparison using the Kruskall-Wallis test or 2 x 3 χ^2^ test.

* Denominator is number of study children who ever had diarrhea.

**Table 3:** Differences in median percentage of days of diarrhea by treatment group and cohort, and for the three cohorts combined.

Treatment group comparison	Cohort		
	HIV-infected children	HIV-uninfected children born to HIV-infected mothers	HIV-uninfected children born to HIV-uninfected mothers
Vitamin A versus vitamin A plus zinc	−2.6 days (−8.8, 2.5)	−0.2 days (−1.3, 0.7)	0.2 days (−0.6, 1.2)
Vitamin A versus multiple micronutrients	−1.9 days (−13.9, 2.8)	−0.2 days (−1.2, 0.7)	0 days (−0.8, 1.1)
Vitamin A plus zinc versus multiple micronutrients	0.1 days (−8.3, 7.1)	0 days (−0.9, 1.2)	0 days (−1.1, 0.8)
	Three cohorts combined		
	Vitamin A versus vitamin A plus zinc	Vitamin A versus multiple micronutrients	Vitamin A plus zinc versus multiple micronutrients
	0 days (−0.8, 0.6)	0 days (−0.5, 0.8)	0 days (−0.8, 0.7)

Values are the difference between groups in median prevalent days of diarrhea (95% CI for the difference). Differences between groups are estimated by calculating the median of all possible differences between patients in the groups being compared, rather than the arithmetic difference in the population median.

#### Respiratory disease morbidity

There were no differences between study groups in the median percentage of weeks with upper respiratory symptoms, which ranged from 13.1% to 16.6% in the three treatment groups in the two cohorts of children without HIV infection; or in the percentage of children who had pneumonia confirmed by measured respiratory rate, which ranged from 4.3% to 19.1% (Table 2).

#### Combined comparison of all cohorts

When the three study cohorts were combined there were no significant differences in either the primary or secondary outcomes by supplement given (Table 4).

**Table 4:** Outcomes of 335 children with at least one home visit by treatment arm for all three cohorts combined

Characteristic	Treatment Group			P
	Vitamin A (n = 113)	Vitamin A+zinc (n = 113)	Multiple micronutrients (n = 109)	
**Diarrhea morbidity**				
Percentage of days with diarrhea per child	2.3 (0.8–4.5)	2.4 (0.7–5.5)	2.5 (0.8–5.4)	0.935
Children who ever had diarrhea, n (%)	98 (86.7)	92 (81.4)	89 (81.7)	0.484
Children who ever had blood in stools, n (%)	26 (23.0)	26 (23.0)	27 (24.8)	0.939
Diarrhea episodes per child	2 (1–5)	3 (1–4)	2 (1–5)	0.917
Duration of diarrhea episodes, days	3.0 (2.5–4.6)	4.0 (2.7–5.0)	4.0 (2.6–5.7)	0.067
Children who ever had diarrhea episodes ≥14 days, n (%)	5 (4.4)	11 (9.7)	12 (11.0)	0.168
Children with >5 stools/day on worst day of diarrhea episode*	40/98 (40.8)	43/92 (46.7)	43/89 (48.3)	0.550
Children who ever visited clinic for diarrhea, n (%)	61 (54.0)	48 (42.5)	52 (47.7)	0.223
Children ever admitted to hospital with diarrhea: n (%)	2 (1.8)	4 (3.5)	7 (6.4)	0.195
**Respiratory morbidity**				
Percentage of weeks with upper respiratory illness signs per child	15.4 (8.4–24.4)	15.8 (8.0–26.1)	15.7 (8.4–25.0)	0.695
Children who ever had pneumonia by maternal report, n (%)	23 (20.4)	30 (26.5)	21 (19.3)	0.366
Children who ever had pneumonia confirmed by respiratory rate, n (%)	17 (15.0)	20 (17.7)	11 (10.1)	0.261
**Other outcomes**				
Died during study, n (%)	3 (2.7)	6 (5.3)	3 (2.8)	0.478
Admitted to hospital for any reason during study	4 (3.5)	10 (8.8)	11 (10.1)	0.141

Values are median (25^th^–75^th^ centiles) or n (%). P values are for the overall group comparison using the Kruskall-Wallis test or 2 x 3 χ^2 ^test.

* Denominator is number of study children who ever had diarrhea.

### Ancillary analyses

There was no effect of treatment when subjects 6–12 months or 12–24 months, or those less than 1.5 or 2.0 z scores of length-for age, were analyzed separately.

Study subjects received supplements 77% of days they were enrolled in the study (Table 1), but there was no significant difference between groups in percentage of days supplement was taken. Among the participant characteristics during study that might have confounded study outcome the only significant differences were the median number of study weeks that study subjects were fed cereal, which differed between treatment groups in both the cohorts of HIV-infected children, and HIV-uninfected children born to HIV-infected mothers; and the clinic used by HIV-infected children. Neither of these variables was associated with days of diarrhea, however, and thus we did not adjust for them in the analyses of outcomes.

### Adverse Effects

Vomiting in the 30 minutes after administration of the supplement was witnessed by the field worker 7 times with vitamin A and zinc, and once each following administration of vitamin A and multiple micronutrients.

## Discussion

### Interpretation

In this study neither zinc combined with vitamin A, nor zinc and vitamin A combined with other micronutrients, reduced diarrhea and respiratory disease morbidity in three cohorts of children 6–24 months of age in rural South Africa–HIV-infected children, HIV-uninfected children born to HIV-infected mothers, or children born to HIV-infected mothers. Enrollment of children with HIV infection was much smaller than the proposed sample size in the study protocol, thus limiting any conclusions about efficacy in this cohort. But enrollment in the other two cohorts was approximately what was planned, and conclusions on findings in these cohorts are thus robust.

### Generalizability

The findings of this study are in contrast to the preponderance of findings from previous studies.[6] A meta-analysis of seven studies of continuous supplementation with zinc found a 25% reduction in diarrhea prevalence and 18% reduction in diarrhea incidence,[6] and in the four studies included in the meta-analysis that reported the effect of zinc on pneumonia there was a 41% decrease in pneumonia incidence.[6]

What differences in study population, study design or supplement formulation might have accounted for the lack of effect of zinc or micronutrient supplementation on diarrhea or respiratory morbidity in this study when compared to previous studies? Children enrolled in this trial had better anthropometric indices than those reported on in most previous studies. Despite the food insecurity and poverty in the community, only 6.4% of children in this study were <2 z scores for length-for-age, compared to 32–100% of children in studies included in the meta-analysis of prophylactic zinc supplementation. A number of studies have shown the impact of zinc prophylaxis is greatest on those who are stunted or underweight,[7], [20], [21] or enrolled only those who were undernourished.[22], [23] [24], [25] Studies that have not found an effect on diarrheal or respiratory morbidity have often included better nourished children.[26] Other studies used zinc supplementation as secondary prophylaxis, enrolling children already ill with diarrhea,[24], [27], [28] which may select for a population at greater risk of disease, and more likely to benefit from supplementation.

Diarrhea morbidity in this study was relatively low when compared to some studies showing an effect of zinc, where the reported incidence has been as high as an improbable 30 diarrhea episodes per child per year.[20] Other studies, like ours, have found much lower rates,[7], [8], [29] This suggests that either case definitions, or methods of ascertainment, in addition to biological and epidemiological factors, may play a role in the variation in diarrhea morbidity found in different studies. We used diarrhea case definitions that should be sensitive measures of diarrhea and that incorporated local terminology for diarrheal illness.[17], [30] The same is true for the definition of pneumonia–the commonly used WHO/UNICEF Integrated Management of Childhood Illness definition of pneumonia was used.[5] The schedule of household visits–once per week–is one that is commonly used, and has been sensitive for detecting diarrhea during community-based studies. The incidence and percentage of days with diarrhea was consistent with what was predicted in estimating the sample size.

The dosage of zinc used in this study–10 mg per day for six months–is the regimen that has been most commonly used in prophylactic supplementation studies,[6] and previous studies with this formulation of micronutrients have suggested good bio-availability.[31] The proportion of study days that the supplement was taken–77% across all study participants–was lower than in some other studies, which have achieved adherence rates as high as 90%.[28] This study was, however, conducted as an intention-to-treat study, and thus data from study participants who had stopped taking supplements, but who still agreed to home visits, are included in the analysis. Some studies have only provided supplementation six days per week. Other studies have provided 20 mg of zinc as a weekly supplement in order to improve convenience and adherence. The rate at which supplements were taken–effectively 5 days per week in this study–would in any case likely be the best that could be achieved under non-study conditions in a general population.

There was no placebo group in this study, and it is possible that vitamin A alone was sufficient to reduce diarrhea and respiratory morbidity. Although there is strong evidence for an effect of vitamin A on mortality in developing countries,[32] there is less consistent evidence that vitamin A supplementation alone reduces diarrhea and respiratory morbidity,[33] and some studies have even reported an increase in respiratory morbidity following vitamin A supplementation.[26]

In October 2003, soon after study enrollment began, the South African government implemented regulations requiring the fortification of maize meal and wheat flour, including flour used by bakeries, with zinc and other micronutrients.[34] The amount of fortification (up to 30 mg zinc per kg of maize meal for instance) in these staple foods, although unlikely to provide an amount of zinc equivalent to that in the supplements, may still have improved micronutrient status in children with access to commercially produced meal or bread.[35] A previous study from South Africa, however, suggest that fortification of maize meal, the staple for these children, did not improve zinc nutriture.[36]

There has been hope that supplementation with multiple micronutrients might be more beneficial that supplementation with a single micronutrient, especially as deficiencies in one micronutrient may limit the functional activity of other micronutrients.[1] To date, however, there has been little evidence that the addition of other micronutrients, including formulations similar to that used in this study, enhances the efficacy of zinc in reducing diarrhea and respiratory morbidity when the latter has been found effective. [21],[29],[37],[38],[39],[40] In at least two studies multiple micronutrient supplementation actually increased rates of morbidity when compared to placebo or zinc alone.[21],[28]

None of the studies reported on in the meta-analysis finding efficacy of zinc prophylaxis for diarrhea or respiratory disease was conducted in Africa.[6] Since the meta-analysis was conducted there have been at least five studies in addition to this one conducted in Africa that have examined the effect of zinc on diarrheal and respiratory morbidity, though in none except this one was it a primary study outcome (Table 5).[7], [8], [40]–[42] In a study in Ethiopia zinc significantly reduced the incidence of diarrhea and cough in children who were <2 z scores for length-for-age, but not in those who were better nourished.[7] In a study in Burkina Faso there was a modest but significant 13% reduction in diarrhea prevalence, but no significant reduction in cough.[8] A study in malnourished children in Lesotho of zinc supplementation for three months after hospital discharge found a reduction in diarrhea prevalence from 37% in the placebo to 3% in the zinc-supplemented group, and a reduction in acute respiratory infection from 39% to 3%.[42] A study of multiple micronutrient supplementation in South Africa did not find an effect of a zinc-containing micronutrient mixture on diarrhea morbidity despite very high rates of diarrhea prevalence.[40] A study in children in South Africa infected with HIV who were not on antiretroviral therapy found a reduction in diarrhea prevalence during scheduled or unscheduled clinic visits.[41] A recent large study from Tanzania found no effect of zinc supplementation on overall mortality, or diarrhea-related mortality.[43]

**Table 5:** Studies of zinc supplementation, either alone or with other micronutrients, for prevention of diarrhea and respiratory illness in African children.

Author	Country	Date	Study population	Study method	Primary outcome	Intervention A	Frequency and duration	Intervention B	Frequency and Duration	Nutritional status Group A	Nutritional status Group B	Effect of intervention on diarrhea	Effect of intervention on respiratory illness
Umeta[7]	Ethiopia	2000	Breast-fed infants 6–12 m	Double-blind RCT. Daily home visits	Linear growth	10 mg zinc sulphate. 45 stunted children <2 z score length-for-age; 45 age and sex matched non-stunted children	Daily except Sunday for six months	Placebo. 45 stunted children <2 z score length-for-age; 45 age and sex matched non-stunted children	Daily except Sunday for six months	Z-scores for stunted zinc group: −2.74 for length-for-age; −2.46 for weight-for-age; −0.48 for weight-for-length. For non-stunted group: −0.70 for length-for-age; −1.35 for weight-for-age; −1.00 for weight-for-length	Z-scores for stunted placebo group: −2.87 for length-for-age; −2.70 for weight-for-age; −0.69for weight-for-length. For non-stunted group: −0.57 for length-for-age; −1.45 for weight-for-age; −1.27 for weight-for-length	For stunted group, 13 episodes (0.6 episodes per child per year) in zinc-supplemented group vs. 40 episodes (1.7 episodes per year) in placebo group (P<0.001). For non-stunted group, 14 episodes (0.6 episodes per year) in zinc group, 19 episodes (0.8 episodes per year) in placebo group (P = NS).	For stunted group, 15 episodes of cough (0.7 episodes per child per year) in zinc-supplemented group vs. 30 episodes (1.3 episodes per year) in placebo group (P<0.05). For non-stunted group, 12 episodes (0.5 episodes per year) in zinc group, 21 episodes (0.9 episodes per year) in placebo group (P = NS).
Müller[8]	Burkina Faso	2001	Children 6–31 m from DSS	Double-blind RCT. Home visits 2 per week	Reduction in malaria incidence	12.5 mg zinc sulphate. 341 children	Daily except Sunday for six months	Placebo. 344 children	Daily except Sunday for six months	Reported jointly for zinc and placebo groups: 36.3% <2 z score height-for-age; 24.6% <2 z score weight-for-height		1.8% days with diarrhea in zinc group; 2.0% in placebo group. RR 0.87 (95% CI 0.79 to 0.95)	2.0% days with cough in zinc group; 1.9% in placebo group. RR 1.05 95% CI (0.97 to 1.15)
Makonnen[42]	Lesotho	2003	Malnourished children 6–60 months identified in hospital and followed at home after discharge	Double-blind RCT. Monthly home visits after discharge from hospital	Not specified	10 mg zinc sulphate in 150 children	Daily for three months after hospital discharge	Placebo in 150 children	Daily for three months after hospital discharge	91% <2 z score weight-for-age	87% <2 z score weight-for-age	At 90 days, prevalence of diarrhea in zinc-supplemented group was 2.9%; in placebo group was 36.7% (95% CI for difference reported as −32 to −15%).	At 90 days, prevalence of acute respiratory infection in zinc-supplemented group was 2.9%; in placebo group was 38.8% (95% CI for difference −45 to −26%).
Smuts[40]	South Africa	2006	Infants 6–12 m	Double-blind RCT. Weekly home visits	Change in weight-for-age status	Intervention A: 1 daily allowance of multiple micronutrients given daily to 49 children or six months	Intervention B: 2 x daily allowance of multiple micronutrients given weekly, with placebo on other days, given to 46 children for six months	Intervention C: 10 mg elemental iron given daily to 49 children for six months	Intervention D: placebo given daily to 50 children for six months	For total study population, 1.6% <2 z scores for weight for age; 10.7% <2 z scores for length-for-age; 0% <2 z scores for weight-for-length.		Prevalent days of diarrhea were 20.9% in placebo group; 22.7% in daily micronutrient group; 21.1% in weekly micronutrient group; and 17.9% in daily iron group (P = NS)	Prevalent days of upper respiratory illness were 9.0% in the placebo group; 6.8% in the daily micronutrient group; 8.7% with the weekly micronutrient group; and 7.9% in the daily iron group (P = NS)
Bobat[41]	South Africa	2006	HIV infected children 6–60 m not on ART	Double-blind RCT. 7 clinic visits during 6 month supplementation period; 1 visit 3 months after supplementation ended.	Change in plasma HIV-1 RNA	10 mg zinc sulphate to 46 children	Daily for six months	Placebo to 50 children	Daily for six months	Height-for-age z score: −1.5. Weight-for-height z score: −0.05	Height-for-age z score: −1.7. Weight-for-height z score: −0.11	In zinc group diarrhea prevalence 6.7% at 360 scheduled clinic visits: 7.4% at 407 total clinic visit; in placebo group rates 105% and 14.5% respectively (P = 0.001 by logistic regression for all events during all visits).	In zinc group pneumonia prevalence 10.8% at 360 scheduled clinic visits: 14% at 407 total clinic visit; in placebo group rates 12.7% and 18.6% respectively (P = 0.07 by logistic regression for all events during all visits). Rates of upper respiratory infection and ear infection also did not differ significantly.

### Overall evidence

What are the policy implications of this study? Because of the heterogeneity of different populations of children in developing countries and the heterogeneity of the studies of zinc that have been conducted to date, generalizing from any single study is problematic. This study had as its outcomes the efficacy of zinc or multiple micronutrient supplementation as primary prophylaxis of diarrhea and respiratory morbidity. Neither supplement was efficacious for either indication.

The study was conducted in children that were less severely stunted than in most previous micronutrient supplementation studies, and who may have benefited from recent efforts to fortify staple food with micronutrients. But it is still a population which has a high burden of ill-health, with an infant mortality rate of 68 per thousand and an child mortality ratio of 98 per thousand.[44] The findings of this study, along with a previously reported study of micronutrient supplementation conducted elsewhere in South Africa that did not show efficacy,[40] do not provide support for the prophylactic use of zinc or multiple micronutrients to reduce diarrhea or respiratory morbidity in the general population of South African children.

## Supporting Information

Checklist S1 CONSORT Checklist
(0.05 MB DOC)

Protocol S1 Trial Protocol
(0.41 MB DOC)
