# Supplementary material for: Zinc or Multiple Micronutrient Supplementation to Reduce Diarrhea and Respiratory Disease in South African Children: A Randomized Controlled Trial
Source: PLoS One. 2007 Jun 27;2(6):e541. doi: 10.1371/journal.pone.0000541 (PMC1891438; doi:10.1371/journal.pone.0000541)
Supplement: Protocol S1 — Trial Protocol (0.41 MB DOC) [file pone.0000541.s002.doc]

# Micronutrients and enteric infections in African children

# The effect of prophylactic micronutrient supplementation on morbidity and growth in HIV-infected and HIV-uninfected children in South Africa

**A Single Center Study of the NIAID**

New England Medical Center/Tufts University

University of Natal/Africa Centre, South Africa

International Collaboration in Infectious Disease Research

Sponsored by:

**The National Institute of Allergy and Infectious Diseases**

**International Centers for Tropical Disease Research Network**

Principal Investigator: ML Bennish

Africa Centre for Health and Population Studies

PO Box 198, Mtubatuba, 3935,

South Africa

*Tel: 27 (35) 550 7502 / Fax: 27 (35) 550 7565*

mbennish@africacentre.ac.za

Major Foreign Collaborator: JME Van den Broeck

Africa Centre for Health and Population Studies

Tel:27 (35 (550-7546 / FAX: 27 (35) 550 7565

Jan.broeck@africacentre.ac.za

Address for express mail:

Africa Centre

47/48 Jan Smuts Avenue

Mtubatuba, 3935

South Africa

NICHD Protocol Representative: J Read

**Version 3**

**30 May 2005**

# Team Roster

**Principal Investigator**

ML Bennish, Professor and Director

Africa Centre for Health and Population Studies

PO Box 198, Mtubatuba, 3935

South Africa

***Tel: 27 (35) 550 7502***

***Fax: 27 (35) 550 7565***

[mbennish@africacentre.ac.za](mailto:mbennish@africacentre.ac.za)

**Major Foreign Collaborator**

JME Van den Broeck,

Africa Centre for Health and Population Studies

PO Box 198

Mtubatuba, 3935

South Africa

***Tel: 27 (35) 550 7546***

***Fax: 27 (35) 550 7565***

[Jan.Broeck@africacentre.ac.za](mailto:Jan.Broeck@africacentre.ac.za)

**Co-Investigators**

N Rollins

Africa Centre for Health and Population Studies

PO Box 198

Mtubatuba, 3935

South Africa

***Tel: 27 (31) 260 4415***

***Fax: 27 (35) 550 7565***

[rollinsn@africacentre.ac.za](mailto:nrollins@africacentre.ac.za)

D.McGrath

Africa Centre for Health and Population Studies

PO Box 198

Mtubatuba, 3935

South Africa

***Tel: 27 (31) 260 4451***

***Fax: 27 (15) 260 4543***

[dmcgrath@africacentre.ac.za](mailto:dmcgrath@africacentre.ac.za)

HD Ward, Assistant Professor

Division of Geographic Medicine

and Infectious Diseases

New England Medical Center

Box 041, 750 Washington Street

Boston, MA 02111

***Tel: (617) 636 7032***

***Fax: (617) 636 5292***

[hward@lifespan.org](mailto:hward@lifespan.org)

HM Coovadia, Professor

HIV Research Institute,

University of Natal, Durban

Private bag 7, Congella 4013,

South Africa

***Tel: 27 (31) 260 4345***

***Fax: 27 (31) 260 4388***

[coovadiah@nu.ac.za](mailto:coovadiah@nu.ac.za)

AW Sturm, Professor and Head

Department of Medical Microbiology

University of Natal, Durban

Private bag 7, Congella 4013, South Africa

***Tel: 27 (31) 260 4395***

***Fax: 27 (31) 260 4431***

[sturm@nu.ac.za](mailto:sturm@nu.ac.za)

S Cassol, Senior HIV/AIDS Scientist

Africa Centre Virology laboratory,

Medical Research Council

PO Box 17120, 771 Umbilo Road,

Congella 4013, Durban, South Africa

***Tel: 27 (31) 204 3600***

***Fax: 27 (31) 204 3601***

[sharon.cassol@mrc.ac.za](mailto:sharon.cassol@mrc.ac.za)

AD Steel, Professor and Director

MRC/MEDUNSA Diarrhoeal Pathogens Research Unit

PO Box 173, MEDUNSA 0204,

Pretoria, South Africa

***Tel: 27 (12) 521 4117***

***Fax: 27 (31) 521 5938***

[adsteele@medunsa.ac.za](mailto:adsteele@medunsa.ac.za)

**NICHD Protocol Representative**

J Read, Pediatric, Adolescent, and Maternal AIDS (PAMA) Branch

National Institute of Child Health and Human Development (NICHD)

National Institutes of Health (NIH)

Executive Building, Room 4B11F

6100 Executive Boulevard MSC 7510

Bethesda, MD 20892-7510

***Tel: (301) 496 7339***

***Fax: (301) 496 8678***

[jr92o@nih.gov](mailto:jr92o@nih.gov)

**Clinical Trials Specialist**

Not Applicable

**Protocol Statistician(s)**

WM Rand, Professor of Biostatistics

Department of Family Medicine and Community Health

Tufts University School of Medicine

136 Harrison Avenue

Boston, Massachusetts 02111

***Tel: (617) 636 0475***

***Fax: (617) 636 4017***

[william.rand@tufts.edu](mailto:william.rand@tufts.edu)

**Protocol Data Manager**

C. Govender,

Africa Centre for Health and Population Studies

PO Box 198

Mtubatuba, 3935

South Africa

***Tel: 27 (35) 550 7512***

***Fax: 27 (35) 550 7565***

[cgovender@africacentre.ac.za](mailto:cgovender@africacentre.ac.za)

**Protocol Epidemiologist**

J Van den Broeck

Africa Centre for Health and Population Studies

PO Box 198

Mtubatuba, 3935

South Africa

***Tel: 27 (35) 550 7546***

***Fax: 27 (35) 550 7565***

[Jan.Broeck@africacentre.ac.za](mailto:Jan.Broeck@africacentre.ac.za)

**Protocol Pharmacologist**

Not applicable

**Community Advisory Board Liaison**

D Biyela, Community Liaison Office

Africa Centre for Health and Population Studies

PO Box 198

Mtubatuba, 3935

South Africa

***Tel: 27 (35) 550 7514***

***Fax: 27 (35) 550 7565***

[duduzile.biyela@africacentre.ac.za](mailto:duduzile.biyela@africacentre.ac.za)

**Regulatory Specialist**

Not applicable

**Pharmaceutical Company Representative(s)**

Not Applicable

**Collaborating Investigators**

AT Tomkins, Professor and Head

Centre for International Child health, Institute of Child Health, 30 Guilford Street,

London WC1N 1EH, England

**Tel: 44 (171)905 2122**

**Fax: 44 (171) 404 2062**

[cich@ich.ucl.ac.uk](mailto:cich@ich.ucl.ac.uk)

# Participating Site(s)

Africa Centre for Health and Population Studies

PO Box 198

Mtubatuba, 3935,

South Africa

***Tel: 27 (35) 550 7509***

***Fax: 27 (35) 550 7565***

The Africa Centre for Health and Population Studies is a collaboration between the University of Natal, Durban, the South African Medical Research Council, and the University of Durban, Westville.

##### Field location

Mpukunyoni Tribal Area,

Umkhanyakude District,

KwaZulu Natal

South Africa

##### Laboratory work

- Africa Centre Virology and Bacteriology Laboratories,
  Sharon Cassol and Wim Sturm (details above)
- Department of Chemical Pathology, University of Natal, Durban,
  Private Bag 7, Congella 4103, South Africa.
  Inga Elson, Dip Clin Path.
  ***Tel: +27 (0)31 260 4365
  Fax: +27 (0)31 260 4517***
  E-mail: [elson@nu.ac.za](mailto:elson@nu.ac.za)
- Centre for International Child Health, London, Andrew Tomkins (details above)

**Table of contents**

Section Page

[**Team Roster 2**](#__RefHeading___Toc527421447)

Participating Site 4

[Study management 9](#__RefHeading___Toc527421449)

[Schema 10](#__RefHeading___Toc527421450)

[1.0 Introduction 11](#__RefHeading___Toc527421451)

[**1.1** **Background** 11](#__RefHeading___Toc527421452)

[**1.2** **Rationale** 11](#__RefHeading___Toc527421453)

[**1.3** **Hypotheses** 13](#__RefHeading___Toc527421454)

[**1.4** **Study Design** 13](#__RefHeading___Toc527421455)

[2.0 Study objectives 15](#__RefHeading___Toc527421456)

[**2.1** **Primary** 15](#__RefHeading___Toc527421457)

[**2.2** **Secondary** 15](#__RefHeading___Toc527421458)

[**2.3** **Tertiary** 15](#__RefHeading___Toc527421459)

[3.0 Selection and enrollment of subjects 15](#__RefHeading___Toc527421460)

[**3.1** **Study Site and Population** 15](#__RefHeading___Toc527421461)

[**3.2** **Inclusion Criteria** 16](#__RefHeading___Toc527421462)

[**3.3** **Exclusion Criteria** 16](#__RefHeading___Toc527421463)

[**3.4** **Enrollment Procedures** 16](#__RefHeading___Toc527421464)

[**3.5** **Sub-studies** 19](#__RefHeading___Toc527421465)

[**3.6** **Co-enrollment Guidelines** 19](#__RefHeading___Toc527421466)

[4.0 Clinical and laboratory evaluations 19](#__RefHeading___Toc527421467)

[**4.1** **Pre-entry/Entry Evaluations** 19](#__RefHeading___Toc527421468)

[**4.2** **Evaluations During Study** 20](#__RefHeading___Toc527421469)

[**4.3** **Evaluations at the Time of Permanent Protocol Discontinuation** 21](#__RefHeading___Toc527421470)

[**4.4** **Post-Protocol Evaluations:** 22](#__RefHeading___Toc527421471)

[**4.5** **Off-Drug Requirements** 22](#__RefHeading___Toc527421472)

[5.0 Data collection and monitoring and adverse experience reporting 22](#__RefHeading___Toc527421473)

[**5.1** **Records to Be Kept** 22](#__RefHeading___Toc527421474)

[**5.2** **Role of Data Management** 23](#__RefHeading___Toc527421475)

[**5.3** **Regional Monitoring** 23](#__RefHeading___Toc527421476)

[**5.4** **Serious Adverse Experience Reporting** 23](#__RefHeading___Toc527421477)

[6.0 Study treatment or intervention 23](#__RefHeading___Toc527421478)

[**6.1** **Study Agent, Administration and Duration** 24](#__RefHeading___Toc527421479)

[**6.2**  **Drug Formulation** 24](#__RefHeading___Toc527421480)

[**6.3** **Study Agent Supply, Distribution and Pharmacy** 24](#__RefHeading___Toc527421481)

[**6.4** **Compliance Monitoring** 24](#__RefHeading___Toc527421482)

[**6.5** **Concomitant medications** 24](#__RefHeading___Toc527421483)

[**6.6**  **Future provision of micronutrient supplements** 25](#__RefHeading___Toc527421484)

[**6.7** **Toxicity Management** 25](#__RefHeading___Toc527421485)

[**6.8** **Criteria for Treatment Discontinuation** 26](#__RefHeading___Toc527421486)

[7.0 Statistical considerations 26](#__RefHeading___Toc527421487)

[**7.1** **General Design Issues** 26](#__RefHeading___Toc527421488)

[**7.2** **Endpoints** 26](#__RefHeading___Toc527421489)

[**7.3** **Sample Size and Accrual** 28](#__RefHeading___Toc527421490)

[**7.4** **Monitoring and Analysis** 29](#__RefHeading___Toc527421491)

[**7.5** **Limitations** 29](#__RefHeading___Toc527421492)

[8.0 Human subjects 30](#__RefHeading___Toc527421493)

[**8.1** **Institutional Review Board (IRB) Review and Informed Consent Process** 30](#__RefHeading___Toc527421494)

[**8.2** **Subject Confidentiality** 30](#__RefHeading___Toc527421495)

[**8.3** **Study Discontinuation** 30](#__RefHeading___Toc527421496)

[9.0 Plans for distribution of research findings to study community 30](#__RefHeading___Toc527421497)

[10.0 Biohazard containment 31](#__RefHeading___Toc527421498)

[**10.1 Specimen handling and shipping** 31](#__RefHeading___Toc527421499)

[11.0 References 32](#__RefHeading___Toc527421500)

[Appendices 33](#__RefHeading___Toc527421501)

[Appendix I Schedule of Events 33](#__RefHeading___Toc527421502)

[Appendix II Information Sheet and Consent Form 39](#__RefHeading___Toc527421503)

[Appendix III Adverse Events 43](#__RefHeading___Toc527421504)

[Appendix IV Community Advisory Boards 45](#__RefHeading___Toc527421505)

[Appendix V Laboratory Methods 46](#__RefHeading___Toc527421506)

[Appendix VI Grading of Skin Reactions for nevirapine related reports 49](#__RefHeading___Toc527421507)

[Appendix VII Forms 55](#__RefHeading___Toc527421509)

[Appendix VIII Multiple micronutrient supplementation 56](#__RefHeading___Toc527421510)

# Study management

All questions concerning this protocol should be sent via e-mail to:

JME Van den Broeck, MD, PhD

Africa Centre for Health and Population Studies

PO Box 198

Mtubatuba, South Africa

***Tel: 27 (35) 550-7546***

***Fax: 27 (35) 550-7565***

[Jan.Broeck@africacentre.ac.za](mailto:Jan.Broeck@africacentre.ac.za)

For questions about inclusion/exclusion criteria, schedule of events, case report forms, entering waivers for randomization/registration exemptions, transfers, and delinquencies, and other data management issues, Dr. JME Van den Broeck will respond:

Send an e-mail message to [Jan.Broeck@africacentre.ac.za](mailto:Jan.Broeck@africacentre.ac.za)

Include the patient ID and study ID

Give a detailed description of the question

**FOR AER QUESTIONS:**

Send an e-mail message to [jan.Broeck@africacentre.ac.za](mailto:jan.Broeck@africacentre.ac.za)

call: JME Van den Broeck

Tel: +27 (35) 550 7546

# Schema

The effect of prophylactic micronutrient supplementation on morbidity and growth in HIV-infected and HIV-uninfected children in South Africa

COUNTRY: South Africa

ESTIMATED COMPLETION DATE: June 2005

DESIGN: Randomized, double blind, clinical controlled trial

SAMPLE SIZE: ***516 children***

POPULATION: HIV-infected children; HIV-uninfected children of HIV-infected mothers and HIV-uninfected children of HIV-uninfected mothers from the Mpukunyoni area of Hlabisa District, KwaZulu Natal, South Africa

STRATIFICATION: a. HIV-infected children

b. HIV-uninfected children of HIV-infected mothers

c. HIV-uninfected children of HIV-uninfected mothers

REGIMEN: a) Vitamin A
b) Vitamin A + zinc

c) Multiple micronutrients including vitamin A + zinc

Prophylactic regimen from 6 months until 24 months of age

Objectives:

Primary objective

To compare the effect of three micronutrient supplements;

1. vitamin A only;

b) vitamin A and zinc;

c) a micronutrient mixture containing vitamins A, B, C, D, E, K, and calcium, copper, folate, iodine, iron, magnesium and zinc;

on prevalent days of diarrhea in three groups of children;

1. HIV-infected children
2. HIV-uninfected children born to HIV-infected women;

c) HIV-uninfected children born to women without HIV infection

Secondary objectives

1. to describe the pathogen-specific pattern of enteric infections in both HIV-infected and uninfected children during ages 6-24 months, with a focus on infection with *C. parvum* and other protozoan pathogens
2. to conduct a cost-effectiveness analysis of micronutrient supplementation in children aged 6-24 months

Tertiary objectives

1. to assess whether either zinc alone or a micronutrient mixture containing zinc affects linear growth and body composition
2. to determine if infection with specific enteric pathogens is associated with the development of persistent diarrhoea lasting >14 days.

## 1.0 Introduction

### **1.1 Background**

Enteric infection and diarrhea remain, with acute respiratory tract infections, the leading causes of death of children in developing countries. Much of the impact of diarrheal illness on health is mediated by its effect on nutrition. Among children infected with human immunodeficiency virus type 1(HIV), diarrhea is thought to be more common, more severe, and more prolonged than in children who are not HIV-infected. Efforts to reduce the prevalence of diarrhea in HIV-infected children, and better manage those episodes of diarrhea that do occur, may both extend and improve the quality of life. Because multi-drug anti-retroviral therapy is currently not accessible for most African children, alternative means of achieving these goals must be sought.

The HIV epidemic has been superimposed upon pre-existing nutritional deficiencies in many countries. The diet among poor South African children consists largely of maize porridge and occasional vegetables, and is often deficient in protein. Because of this limited diet, vitamin A and zinc are likely to be deficient in South African children, particularly amongst HIV-infected children, though local studies have not been conducted to confirm this. Vitamin A has long been known to be important for maintenance of epithelial integrity and function, including gut mucosa.

Zinc is also important in the control of growth and repair of intestinal mucosa.(2) Mild zinc deficiency has been associated with villus atrophy and reduced brush border disaccharide activity.(3) Clinical studies of the effect of zinc given either prophylactically or therapeutically have demonstrated a 32.5% reduction in diarrheal duration with zinc supplementation in children with low rectal mucosal zinc levels, and a 9.3% reduction in all children.(4) These findings have been confirmed in large community-based studies in India (5) and amongst malnourished children in Bangladesh (6). Smaller studies of the efficacy of zinc supplementation have also been carried out at additional sites in India, and in Vietnam and Mexico (7-10). No comparable data are currently available in African children. No studies to date have been conducted in HIV-infected children, among whom both persistent and recurrent diarrhea, and trace element deficiencies may be common.

Surprisingly little is known about patterns of enteric infection in HIV-infected children, its effect on their health and nutrition, and its relationship to HIV activity and host immune status. Common enteric pathogens such as rotavirus appear to be a frequent cause of acute diarrhea,(11-14) although geographic variation may exist.(15,16) In Durban, South Africa, C. *parvum* was detected in 24.8% of HIV-infected children presenting with persistent and was associated with a lower CD4 count and higher mortality.(17) Particularly little is known about the role of the newly recognized enteric pathogens *C. parvum, Cyclospora cayetanensis,* *Enterocytozoon bieneusi* and *Mycobacterium avium* in childhood in Africa, and especially in AIDS-associated diarrhea.

The study described below will assess the benefit of prophylactic zinc and micronutrient supplements given to both HIV-infected and uninfected infants for 18 months on the prevention of diarrhea and on improvements in linear growth, weight gain, and lean body mass. Also, the infective agents responsible for diarrhea in these children and pathogen-specific effects on nutritional status will be investigated until 2 years of age. This will allow us to determine the impact of specific pathogens on progression to chronic diarrhea.

### **1.2 Rationale**

A major initiative to reduce the impact of enteric infections on health and nutrition has been micronutrient supplementation. Measures for routine vitamin A and zinc supplementation are gaining increasing support from international agencies, including UNICEF and the WHO. Information on the utility of supplementation with these micronutrients, and especially supplementation with zinc, come largely from studies conducted in the Indian sub-continent and other regions of Asia, regions that differ from sub-Saharan Africa in a number of important characteristics. There is little information on the potential utility of micronutrient supplementation on infection and health of African children. Correlation of enteric pathogens, particularly C. *parvum* genotypes, with epidemiological (drinking water source, contact with domestic animals, etc), and clinical features (HIV status, CD4 counts, viral load, severity of illness) may provide important information such as source of infection, route of transmission, and susceptibility of HIV-infected children to infection with a particular pathogen.

In addition to children with HIV infection, two groups of HIV-uninfected children will also be enrolled in this proposed study – those born to HIV-infected mothers, and those who are born to HIV-uninfected mothers. Uninfected children born to HIV-infected mothers are likely to resemble HIV-infected children in their socioeconomic characteristics. Children of HIV-uninfected mothers are included for two reasons. First, the care practices of mothers who have HIV infection may differ from mothers without HIV. This may alter the infants’ nutritional status and predispose to enteric and other infections. Secondly, and more significantly, HIV-infected mothers whose children are uninfected at 4 months will be advised to stop breastfeeding completely as soon as possible after the child has reached 6 months of age. In contrast, uninfected mothers will be advised to continue breastfeeding until the child is at least two years old. This difference in feeding practice may have an important effect on the pattern of enteric infections in the first two years of the child’s life. In any case, despite the very high rate of HIV infection in this community (approximately 35% of all pregnant women), the majority of women are not HIV-infected, and it is important to determine the potential utility of micronutrients in these children.

We will have three treatment groups in this study – a group who receives vitamin A alone, a group that receives vitamin A and zinc, and a group that receives vitamin A, zinc, and multiple micronutrients. Vitamin A in a dose of 1250 IU daily will be given as a control in one arm of the study. We decided to do this because periodic vitamin A supplementation (100,000 IU as retinol palmitate given orally every three months between 6 and 12 months, and 200,000 units every three months between 12 and 60 months) is recommended in the current Integrated Management of Childhood Illness (IMCI) guidelines in South Africa. Although this is not actually done in practice in Hlabisa District, and the evidence for its efficacy in this population is limited, we felt it would be remiss to not provide vitamin A as part of the study. In a local study, vitamin A has been demonstrated to reduce morbidity in young children infected with HIV. (18)

Although the policy in place in South Africa is to give vitamin A periodically in large doses, for purposes of this study we felt it better to give it in smaller daily doses. The basis for this massive dosing of vitamin A at 3 monthly intervals is based on programmatic rather than physiological considerations. Following this massive dosing schedule in the control group in our study would not allow for blinding. If the nutrients provided in the intervention group – such as iron and zinc - were given at three monthly intervals in amounts equivalent to the massive vitamin A administration severe toxicity might ensue. For this reason it is impossible to match the massive vitamin A dosing schedule with an equivalent micronutrient preparation given 3 monthly. As the intervention study group is to receive micronutrients on a daily basis, we decided that it was more appropriate, and physiological, to give vitamin A on a daily basis.

In contrast to vitamin A supplementation, iron supplementation is not part of current IMCI recommendations, and we will thus not give routine iron supplementation. We will provide iron supplementation to those children who on routine testing have hemoglobin concentrations less than 8.0 mg/ml. The supplementation will be approximately 2mg/kg for 12 weeks, at which time the hemoglobin concentrations will again be determined, and iron supplementation discontinued if the iron hemoglobin concentrations are 10 mg/ml or above. This scheme is similar to that advised for populations where anaemia is highly endemic in southern Africa and also in the Handbook of Clinical Management Protocols in use at Hlabisa Hospital.

All micronutrient supplementations will be provided on a daily basis. The decision to provide daily supplementation, rather than semi-weekly supplementation, was made in discussions at the NIH Child Micronutrient Meeting held in Bethesda, December 8-9, 2000. Those discussions suggested that daily supplementation would be a more efficacious regimen given the pharmacokinetics of some trace elements and the water-soluble vitamins and is also in keeping with interventions planned in other international studies

We will provide zinc supplementation at 10 mg elemental zinc per day. Though larger supplements have been used, there is concern that excess zinc supplementation may impair immune response. Malnourished children in Bangladesh who received 6mg/kg/day had a significantly higher mortality than those who received 2 mg/kg/day.

The concentrations of the micronutrients in the multiple micronutrient supplementation were those recommended by a joint UNICEF/WHO meeting held in October, 1999 in New York to develop guidelines for testing of multiple micronutrient supplementation. We have followed these guidelines in order to be consistent with other proposed studies. We have not included selenium in the micronutrient mixture to be used in thus study because of unresolved issues of product preparation. Should this issue be successfully resolved before the start of the study we will amend the protocol to include it.

Micronutrient interventions represent an affordable option for most countries in Africa. If one of the formulations evaluated in this study is found to be effective in reducing morbidity, it would provide a rationale for large scale interventions. Such large scale interventions could bring the costs down even further. This study will carry out a cost-effectiveness analysis to document this. Given the similarities of the study site to other locations in Africa, the findings will have a direct bearing throughout sub-Saharan Africa and may profoundly impact on the care, comfort and lifestyles of mothers and children infected with HIV.

### **1.3 Hypotheses**

##### Main hypothesis

Prophylactic multiple micronutrient supplements act synergistically with zinc to reduce the prevalent days of diarrhea in both HIV-infected and uninfected children as compared to vitamin A alone.

##### Secondary hypotheses

1. Pathogen-specific patterns of enteric infections differ between children who take daily vitamin A supplements and children who take daily vitamin A plus zinc or multivitamins and minerals
2. Total cost of child illnesses between 6-24 months is not different between children who take daily vitamin A supplements and children who take daily vitamin A plus zinc or multivitamins and minerals

### **1.4 Study Design**

A randomized, double blind, clinical controlled trial with three arms in a study population of infants, stratified by HIV status and the HIV status of their mothers.

| HIV status | **Vitamin A**  **1250 IU**  **daily** | **Vitamin A 1250 IU,**  **+ zinc 10mg daily** | **Vitamin A, 1250 IU,**  **+ zinc 10 mg,**  **+ micronutrient mixture daily** |
| --- | --- | --- | --- |
| *HIV-infected children* | * | * | * |
| *HIV-uninfected children of HIV-infected mothers* | * | * | * |
| *HIV uninfected children of uninfected mothers* | * | * | * |

## 2.0 Study objectives

### **2.1 Primary**

To compare the effect of three micronutrient supplements;

1. vitamin A only;
2. vitamin A and zinc;
3. a micronutrient mixture containing vitamins A, B, C, D, E, K, and calcium, copper, folate, iodine, iron, magnesium and zinc;

on prevalent days of diarrhea in three groups of children;

1. HIV-infected children
2. HIV-uninfected children born to HIV-infected women;
3. HIV-uninfected children born to women without HIV infection

### **2.2 Secondary**

1. to describe the pathogen-specific pattern of enteric infections in both HIV-infected and uninfected children during the first 24 months of life, with a focus on infection with *C. parvum* and other protozoan pathogens.
2. To compare total cost of illness 6-24 months between the three treatment arms in each of the three cohorts of children.

### **2.3 Tertiary**

1. to assess whether either zinc alone or a micronutrient mixture containing zinc affects linear growth and body composition.
2. to determine if infection with specific pathogens is associated with the development of persistent diarrhea lasting >14 days among those enrolled in any of the arms of the study.

## 3.0 Selection and enrollment of subjects

### **3.1 Study Site and Population**

The proposed study will be conducted through the Africa Centre for Health and Population Studies located in Hlabisa Health District, in northern KwaZulu Natal (KZN), South Africa. The Health District is a rural district where people rely on subsistence farming, migrant labor, and pension remittances from the South African government. In 1996, 22% of women attending antenatal clinics in KZN Province were HIV-infected by ELISA testing compared to 10.4% for the rest of the country. In 2000, the proportion of pregnant women who were infected with HIV had risen to 36.2% in KZN and 24.5% in the whole of South Africa.

The population for this proposed study will consist of children born during a 2 year period in the Provincial Clinics and Hospital in Hlabisa District. The majority of mothers in this area deliver in the local clinics; there are relatively few home births.

- The study will partly follow-on from another study also being implemented at the Africa Centre. The Vertical Transmission Study (VTS) will examine the utility of different feeding regimens in reducing perinatal HIV transmission. Additional risk factors for vertical transmission will also be examined. In this study all pregnant women in the District will be offered HIV testing. Those who are HIV infected will be offered nevirapine (to both the mother and child) in the peripartum period. All children born to HIV-infected women will then be followed, with plasma HIV RNA concentrations determined at birth, 6, 10, 14, 18, 22 weeks and 6 months of age. This testing will be done using dried blood spot samples which will be analyzed in the Africa Centre HIV Laboratory in Durban. In addition, a cohort of HIV-uninfected women is included in the VTS and will form the pool from which the HIV-uninfected children from uninfected mothers in this study will be recruited. HIV-uninfected women are recruited to the VTS for three reasons: a) to compare adherence to exclusive breastfeeding between HIV-infected and uninfected women who receive the same breastfeeding support strategy, b) as a ‘diversion’ so that participation in the VTS does not, by simple association, disclose the HIV status of women within the community, and c) to describe the influence of exclusive breastfeeding on morbidity and mortality in an HIV-uninfected population. The sample size for this study is 2,100 HIV-infected mothers and their exposed infants and 400 HIV-uninfected mothers and infants.

The VTS will follow UNAIDS/WHO feeding recommendations for infants after 6 months of age. WHO recommends exclusive breastfeeding up to 6 months of age. The WHO definition of exclusive breastfeeding allows for the administration of medicines, in addition to breastmilk but not other fluids or foods. After 6 months of age other complementary feeds should be introduced to all children, but breastfeeding is still generally recommended until about 2 years of age. This is internationally accepted best practice for HIV-uninfected women and women of unknown status. HIV-uninfected mothers will therefore be encouraged to continue breastfeeding until their infants are 2 years of age. Similarly, HIV-infected mothers whose children are already HIV-infected at 6 months of age will be encouraged to continue breastfeeding until their infants are 2 years of age. These mothers will be actively supported to do so by the research team. HIV-infected mothers but whose children are HIV-uninfected at 6 months of age will be encouraged to provide a replacement milk where it is safe and feasible to do so. All mothers will be similarly advised regarding complementary feeds.

Eligible children from outside the Vertical Transmission Study can be enrolled in principle but they will only be recruited actively for that purpose if recruitment rates in the transmission study are slower than anticipated. These additional children will then undergo the same diagnostic testing, feeding counseling and intensity of follow-up as the children in the transmission study.

### **3.2 Inclusion Criteria**

- 4-6 months of age (stratified by HIV status)
- Infant able to take oral preparations
- Consent of parent/guardian
- HIV status obtained from mother and child

### **3.3 Exclusion Criteria**

- Documented micronutrient supplementation other than vitamin A in the preceding month
- Less than 60% of mean weight for age by NCHS guidelines (micronutrient intervention obligatory according to WHO guidelines for management of severely malnourished children)
- Persistent diarrhea (> 7 days) at the time of study enrollment
- Exclusive breastfeeding
- Infants in whom a second confirmatory HIV test cannot be obtained (when required)
- Co-enrollment of the infant in other clinical intervention trials e.g. antibiotic or vaccine trials

### **3.4 Enrollment Procedures**

3.41 Determining HIV status

As part of the VTS dried blood spot samples will be collected at birth, 6, 10, 14, and 18 weeks and at 7, 8, 9, 12, 15, 18, 21 and 24 months of age. These samples will be tested for HIV using Organon Teknika quantitative HIV RNA assay. This assay has a detection level of 1600 copies per 50 μl sample. This is equivalent to 80 copies per ml.

3.42 HIV diagnostic algorithm

- *Samples collected at 6 and 18 weeks will be tested real time with Organon Teknika assay.*
- *If HIV RNA not detectable (<80 copies/ml), then the assessment is NEGATIVE ASSAY (NOT HIV-INFECTED)*
- *If 80-10,000 copies/ml, an additional sample will be obtained from the child >72 hours after the initial sample. If this second sample is >10,000 copies/ml the child will be classified as HIV-infected. If < 10,000 copies/ml the child will be classified as HIV-uninfected.*
- *If the initial sample is >10,000 copies/ml a second sample will be obtained. If there are >10,000 copies/ml in the second sample then the child will be classified as HIV-infected, and the parents or guardians informed accordingly. In the rare event that the second sample has a concentration < 10,000 copies/ml, a third sample will be obtained. Classification will then be based on the results of this third sample (< 10,000 copies/ml, HIV-uninfected; > 10,000 copies/ml, HIV-infected).*

Children on whom a second sample cannot be obtained (when required) will be excluded from participation in the study.

This algorithm for diagnosis is consistent with that recommended by the Ghent International Working Group on Mother-To-Child Transmission of HIV. Because we will also obtain samples for HIV RNA testing at 9, 12, 15, 18 and 24 months the validity of this working definition of status can be confirmed. In limited studies to date we have not found false positives with the Organon Teknika assay in children.

3.43 Recruitment, Enrollment and Randomization

* Recruitment strategy

Recruitment of children in the Vertical Transmission Study will be done when the child comes for the 18-week routine clinic visit. Interested mothers will be given an appointment with the study nurses of the Micronutrients Study for evaluation of eligibility criteria and possible enrollment before 26 weeks. Project nurses will be provided every week with a list of potential recruits and appointments.

The recruitment strategy of children from outside the Vertical Transmission Study will include home visits by field staff to mothers with young infants. During these visits mothers will be informed about the existence of the study and asked whether they would like to learn more about the study in view of a possible enrollment in case the child were eligible. In case mothers are interested to learn more they will be offered an appointment with the study nurses of the Micronutrients Study for an enrollment visit to the nearest clinic. The informed consent procedure will than be started during these enrollment visits by the study nurses. All eligible children will be offered enrolment, independent of their HIV status or the HIV status of their mother. The Africa Center’s Demographic Information System (ACDIS) and its HIV Surveillance sub-study will be used as a framework to identify homes with young infants to be visited. ACDIS keeps track of pregnancies and new births in the area and offers VCT (HIV Voluntary Counseling and Testing) to adults. Under no circumstance will only households with HIV-infected mothers be visited. It will be ensured that amongst households visited, there will be at least 15% with HIV-uninfected mothers. Within that restriction any preponderance according to HIV status will be set according to the necessity to speed up the enrollment in a particular cohort. In all cases an anonymous list of homestead identification numbers will be extracted from the ACDIS database, allowing locating the house. Fieldworkers approaching the houses will not know the name of the individual on the basis of whom the homestead was sampled, nor will they have any information regarding the HIV status of the mother or mothers living in these homesteads. As all eligible children will be offered enrollment independent of the HIV status of their mother, there is no risk of disclosure associated with this recruitment strategy. Recruitment outside VTS will be stopped as soon as either the Micronutrients Study cohort of HIV infected mothers and children is full, or, both cohorts of HIV uninfected mothers are full.

* Enrollment

Children who are eligible will be offered enrolment by the study nurses at the clinics until treatment arms are filled. Parents or legal guardians will be advised of the results of HIV testing on their child 2 weeks after the initial sample is obtained, and will be offered counseling regarding the implication of the result. “**When a cohort is full we will continue enrollment in all cohorts in order to maintain community blinding of HIV status of mother and child. However, only 10% of new recruits in the filled cohort will be selected for full treatment and follow-up. The other will undergo reduced treatment (not less than 1 month of tablets) and will receive less frequent home visits (not less than 1 home visit per month). In order to maintain the same level of care, however, the frequency of clinic visits and procedures will remain the same as for other children**”.

A child will only be regarded to have enrolled in the study after:

1. the parent or guardian of the child has been fully informed of the study objectives and methods
2. the child meets the entry criteria.
3. the parent or guardian has given written consent.
4. HIV status of the mother and child availablehas been determined or consent was given for a blood sample for that purpose.

Once consent is obtained, the study staff will phone the study office to communicate core information (name, DOB, name of parent or guardian, address, VTS number).

* Randomization

Randomization to an intervention arm will be managed at the study office at the Africa Centre and be done as soon as HIV testing results of mother and child are available.

1. Children from each of the HIV status groups will be individually randomized to one of the three multivitamin/micronutrient arms (vitamin A; vitamin A + zinc; vitamin A + zinc + multiple micronutrients). Randomization will occur in blocks of 6 within each of the HIV status groups. Randomisation will result in assigning a pre-coded box filled with blister packets of 7 micronutrient tablets each. Each child will use tablets from that same box throughout the study. Each type of tablet will appear and taste identical to ensure blinding of both mothers and field staff. The interval between testing and randomisation may vary depending time required to obtain HIV testing results. All children will, however, start their respective supplement at 6 months of age (±14 days) and will continue with the supplements until they are 24 months of age (i.e. for ~18 months). Monitoring will continue throughout this time to determine the long-term impact of each supplementation. On the first home visit, at 6 months of age, mothers will be shown how to store, prepare and administer the respective supplement. She will also be shown how to keep the morbidity diaries and the fieldworker visiting schedule will be explained in detail. “**A number of randomisation blocks will not be used for randomisation but will be set aside to form a special stock of tablets that can be used for re-assigning new tablet numbers to participants in case a new batch of tablets does not arrive on time. In case of such a discontinuation of stock, and to ensure continuity of treatment, the independent statistician will assign a new tablet number to the participant from this special re-assignment stock, making sure that the chemical composition remains the same and also making sure that full blinding is maintained**”.

3.44 Consent

Parents will be given written information in Zulu (Appendix II) and will have an opportunity to discuss the study with the study nurse or HIV counselor when she/he returns to receive the HIV results of her/his child. If agreeable she/he will be asked to give written informed consent. The information sheet provided with the consent form will include a description of the study, the possible benefits and potential risks of participating in the study, and a statement that participation is entirely voluntary and will not affect the care that the child would otherwise have received. “**When a cohort is full an adapted version of the ICF will be used informing the mother of the possibility that the child will be selected for reduced treatment and follow-up**”

### **3.5 Sub-studies**

None planned up till today

### **3.6 Co-enrollment Guidelines**

Infants will be eligible for other cohort or case control studies but will be unavailable for clinical intervention or vaccine .

## 4.0 Clinical and laboratory evaluations

### **4.1 Pre-entry/Entry Evaluations**

4.11 Mothers and children co-enrolled in the VTS will have been previously tested for HIV and counseled appropriately. They will have been prepared for HIV testing of their child at multiple time points between birth and 24 months of age. Mothers will have been counseled on the nature and purpose of research. Mothers will be informed of this follow-on study and given a written description in Zulu (Appendix II). If a mother needs more time to consider the study or wants to discuss the matter with other family members, an appointment will be given for the following week. Written informed consent will be requested before enrollment.

4.12 A baseline clinical examination, anthropometry assessment and bioelectrical impedance analysis will be performed on each child. Subsequent blood and stool tests will be conducted as per the investigation schedule below.

- 1. **For children not co-enrolled in the Vertical Transmission Study and who are enrolled between 22 and 26 weeks, the 26-week blood sample will be replaced by a single blood sample taken at enrolment. This blood sample will serve for HIV testing, viral load, CD4 count as well as for the baseline biochemical and haematological measurements that, in other children, are done at 26 weeks**”**.**

### **4.2 Evaluations During Study**

4.21 Clinical and laboratory evaluations and methods (see Appendices I & V).

###### 4.211 Blood samples

1. Complete blood count will be measured using standard lab methods. Serum c-reactive protein (CRP) will be measured by established ELISA assays. The CRP measurement will be used to corroborate the validity of serum retinol measurements, which fall rapidly in any acute infection. In order to assess if low levels of retinol are related to a deficient state, serum CRP will be used as a proxy for infection and inflammation. **“CD4 counts will only be measured in HIV infected children”**. “**In case the foreseen amount of blood cannot be obtained, analyses will be prioritised in the following order (1) HIV testing (2) CD4 count and viral load in HIV infected children (3) EDTA-retinol (4) Zinc and CRP**”.

###### 4.212 Stool sampling - routine sampling

###### Children entered into the study will have stool samples obtained at intervals when they do not have diarrhea. All infants will have routine samples collected at 6, 12, 18, and 24 months of age. Samples will be collected from ¼ of the uninfected children at the same times. Such samples will provide information on rates of carriage of enteric pathogens in the absence of diarrhea.

###### 4.213 Stool sampling- during diarrheal episodes

We will attempt to obtain stool samples from all children with diarrhea. We will encourage parents of children with diarrhea to bring their child to the clinic for evaluation. At these visits a stool sample will be obtained if possible. If a child does not produce a stool after a suitable wait (60-120 minutes) a rectal swab sample will be taken. Rectal swab samples will be processed for detection of bacterial and viral pathogens, but not for protozoan pathogens.

Stool containers will also be provided to households of study children, and parents instructed to obtain a stool sample on the day of the field worker’s weekly visit if the child had any new diarrhea episode in the period of 48 hours before the home visit and if the child was not taken to the study nurses for that reason.

###### 4.214 Lean body mass and anthropometry

These will be assessed on study entry at 6 months, and at 12, 18 and 24 months of age, using a RJL bioelectrical impedance analyzer. Specific regression equations are currently being developed using ^18^O and bromide studies for South African children. Body weight, head circumference and length will be determined at the same time as body mass, with weight measured using an electronic scale, head circumference using a tape, and height measured with a stadiometer.

4.22 Clinical events

###### 4.221 Home monitoring

Mothers and children will be visited at home once weekly to ascertain morbidity events and to collect stool samples as per the study schedule. Field workers will visit households on the same days each week, so parents will be able to anticipate their visits. Data will be recorded on forms which will be returned to the study office every week for data entry. (Appendix VII)

###### 4.222 Morbidity diaries

Parents/guardians will be asked to keep a diary of children’s days with diarrhea. This will be a simple daily checkbox on which the parents, or caretakers, will daily record if the child has had diarrhea and if oral rehydration solution has been given. Field workers will use the diary during the interview to ascertain accurate information on days with diarrhea since the last visit. The diary itself will not be captured in the database.

###### 4.223 Fieldworker advice

Field workers will be provided with packets of oral rehydration solution (ORS) to give to families, and will be instructed on how to teach parents to give oral rehydration solution. For dysenteric or febrile diarrhea, field workers will be instructed to tell parents to take their children for care to the local clinics if they have not already done so. Field workers will also provide nutritional advice to parents and other caretakers for these children. Data on usage of ORS will be collected.

###### 4.224 Clinic visits

Mothers will be asked to bring their children to the study clinic at 3 monthly intervals for routine anthropometry measurements and hemoglobin determinations, and bioelectrical impedance analysis of body composition.

4.23 Immunology

The effect of micronutrient supplementation on immune competence will be assessed by measuring CD4 counts.

### **4.3 Evaluations at the Time of Permanent Protocol Discontinuation**

4.31 Subjects who complete the protocol should have the following:

###### 4.311 Clinical Assessments

Children will be assessed in the clinic at enrollment (6 months old), 9, 12, 15, 18, 21 and 24 months. At these visits, children will be assessed clinically and have routine anthropometry taken. Home visits will only obtain a history from the mother, but will not do clinical evaluations except checki9ng for signs of severe dehydration. If the child is unwell the fieldworkers will advise the mother to take the child to a clinic for medical attention.

###### 4.312 Immunology

Samples will be obtained for HIV RNA viral load at enrollment and at 3 monthly intervals thereafter until 24 months of age. CD4 count will be determined as an indicator of immune competence from venous blood samples at 12, 18 and 24 months of age. Additional blood will be taken at these times for a full blood count, serum zinc, tocopherol, retinol, retinol binding protein and CRP.

These will be included as covariates in the regression analyses described in section 7.41

###### 4.313 Parasitology/Virology/Bacteriology

Stool samples will be collected at regular intervals (see 4.212) and also in the event of an acute diarrheal episode (see 4.213). The schedule of tests is outlined in Appendix I and diarrheal pathogens to be examined for are listed in Appendix V.

###### 4.314 Body composition

This shall be assessed at enrollment (6 months of age), and at 12, 18 and 24 months of age.

4.32 Subjects who prematurely discontinue from protocol treatment

###### 4.321 Clinical Assessments

Children who discontinue taking study treatments but who are still accessible, either by continuing to visit clinics or via home visits, will have all scheduled assessments completed. Research staff will conduct an assessment at the home of the child within 2 weeks of the scheduled clinic visit in the event of the mother/ guardian declining to attend the formal clinics.

###### 4.322 Immunology

All samples will be collected as per the schedule if the child is accessible and the parent agrees. A field team will visit the household if the child does not come to clinic and obtain the blood analyses listed in 4.312. This will be done only for children who remain in the District.

###### 4.324 Parasitology / Virology / Bacteriology

A stool sample or rectal swab will be requested by research staff at the time of a final home visit within two weeks of the last failed clinic attendance.

###### 4.325 Body composition

Trained field staff using a portable bioelectric impedance analyzer will assess body composition. This shall be conducted at the time of a final home visit.

### **4.4 Post-Protocol Evaluations:**

Further follow-up of the cohort beyond completion of this study is being considered but will be contingent on funding being secured.

### **4.5 Off-Drug Requirements**

In the event of a death or possible adverse event related to the interventions, the family shall be visited and a “verbal autopsy” or clinical assessment will be undertaken as appropriate by the project physician to determine the likely cause of death or cause of adverse reaction. Biological samples will be requested as deemed appropriate.

After completion of the study, mothers and infants will be followed 3 times each year as part of the routine activities of the Demographic Surveillance System (DSS) of the Africa Centre. The internal study number of infants in this study will be linked with the internal DSS ID number. Verbal autopsies are routinely obtained on persons in the DSS who die. We will be notified and examine the events surrounding the death to determine if there is any possible association with the study interventions.

## 5.0 Data collection and monitoring and adverse experience reporting

### **5.1 Records to Be Kept**

Baseline demographic data will be available from the VTS for co-enrolled children. Morbidity data collected weekly from homesteads will be collected on standard field monitoring forms. These forms will be returned weekly to the Africa Centre study office for capture. Routine growth and unexpected morbidity data collected at the clinics every 3 months will be recorded on forms kept at the clinic for this purpose. The forms will be collected in a folder for each child. These books shall be retained at the clinics to enable clinic staff follow the health of each child.

### **5.2 Role of Data Management**

#### Information system

A single database will serve both this study and the VTS.

Data will be captured by optical image scanning using Teleform (Cardiff Software Inc., San Marcos, CA, USA), a software interface between scanner/FAX and database. Teleform permits several internal validation checks which are incorporated into the database. Following verification, data will be downloaded into a SQL server database. A dedicated database administrator (C Govender) will take prime responsibility for supervising data management in both this study and the VTS.

#### Quality assurance

Field supervisors will check all forms before being submitted for data capture. Discrepancies or missing data will be checked in the field the following day. In addition, a series of field checks are planned to ensure the quality of field monitoring:

1. Planned supervisor visits – supervisors will pre-arrange home visits with the field monitor and will observe the field monitor conduct the interview.
2. Unscheduled supervisor visits – supervisors will make spot checks of field monitors visiting homes and observe the interview.
3. Quality assurance visits – supervisors will visit the same house as the field monitor and on the same day but conduct an interview with the mother/guardian independently. Data will be compared with that of the field monitor. These will be performed on 5% of all visits.

### **5.3 Regional Monitoring**

No independent, external monitoring of the study is currently planned. If the DSMB views this as essential then additional resources will be required to facilitate this activity. We would welcome site monitors contracted to the National Institute of Allergy and Infectious Diseases (NIAID) to visit the site and review the research records for accuracy, completeness and legibility.

Study documents (e.g., consent forms, drug distribution forms, case report forms) and pertinent hospital or clinic records will be made available for inspection.

### **5.4 Serious Adverse Experience Reporting (Appendix III)**

Field staff will document unexpected morbidity episodes and other events not included in the *Field Monitoring Form* on a *Difficulty Report Form*. Suspected serious adverse events will be investigated by a study nurse or doctor to ascertain further details. This information will be recorded on an *Adverse Event Form*. In particular, skin reactions possibly related to nevirapine administration will be ascertained as part of the VTS over the first 3 months of life and up to 2 years of age and graded according to the ranking system used in HIVNET 012, PACTG 316 and HIVNET 023. (Appendix VI) However, by the time infants are enrolled in this study it is extremely unlikely that there will be any adverse event related to nevirapine administered within the first 72 hours of life.

Adverse events will be reported to the DSMB, the Ethics Committee of the University of Natal, and NIAID and NICHD.

## 6.0 Study treatment or intervention

### **6.1 Study Agent, Administration and Duration**

Children of each infection status will be enrolled into one of three arms of the study and daily receive one of the supplements listed below from 6 months until 24 months of age.

| **Intervention arms**  Stratification by HIV status | **Vitamin A**  **1250 IU**  **daily** | **Vitamin A 125 IU + Zinc 10mg daily** | **Vitamin A 1250 IU + Zinc 10 mg + micronutrient mixture daily** |
| --- | --- | --- | --- |
| *HIV-infected children* | * | * | * |
| *HIV-uninfected children of HIV-infected mothers* | * | * | * |
| *HIV-uninfected children of uninfected mothers* | * | * | * |

Supplements will be given as a tablet, which will be identical in appearance and taste to ensure blinding. Tablets will be presented in blister packs containing a supply for one week. Blister packs will be labeled only with a unique code number, which will be allocated to individual children as they enroll into the study. This main supply will be retained at the study office. Mothers will always retain two sets of blister packs to guarantee supply in the event of fieldworkers being unable to complete a visit e.g. inclement weather.

Children will only start receiving supplements after they have started receiving complementary feeds.

### **6.2 Drug Formulation**

All three supplements will be presented using the same powder carrier, stabilizer and flavor. All will have the same taste, appearance, and consistency. The detailed formulation of the multiple micronutrient supplement is described in Appendix VIII. All are made to be crushed and given with food without losing potency.

### **6.3 Study Agent Supply, Distribution and Pharmacy**

6.31 Study supply acquisition

Supplements will be supplied by HERSIL Sa, Av Los Frutales 918, La Molina, Lima, Peru prepared in cooperation with Hoffman-La Roche (Gabriela Lock, HNH Manager, Vitamin Division Hoffmann-La Roche, Peru, Phone: 51 1 4769276, Fax: 51 1 4769273).

### **6.4 Compliance Monitoring**

Trained field workers will visit mothers and children at home once weekly. At the time of each visit the fieldworker will observe the mother/guardian or will herself feed the tablet to the child, either by crushing it into porridge or other spoon feeds, or into milk. Packaging will be collected and the number of tablets given/missing will be recorded.

### **6.5 Concomitant medications**

6.51 Prescribed and over the counter medicines

If an infant receives additional medications, either prescribed by a health worker or purchased over the counter, this information will be documented and captured onto the database, but the infants will not be excluded from the study. Of note, the infant vitamin and mineral supplements locally available in the shops in the study area do not contain zinc.

6.52 District Health System

There is an established and functioning health system in Hlabisa District of South Africa. Basic drugs and treatment protocols are available at all the clinics for most common illnesses. The Maternal and Child Health Directorate of the KwaZulu Natal Provincial Health Authority are training nursing staff throughout the Province in the UNICEF IMCI management and treatment strategy. Hlabisa is included within that training programme.

6.53 Management of diarrhea

Children with diarrhea require treatment early and this immediate support, including oral rehydration and antimicrobials, is readily available at the government health clinics in Hlabisa. The District recently had a very large (and ongoing) cholera epidemic, and managed this very effectively, with extremely low mortality rates. Management of diarrhea is largely based on algorithms that do not incorporate microbiologic data. Information from laboratory cultures in Durban will, however, be returned as soon as they are available, usually 2-3 days after they are obtained. Treatment will be modified accordingly. In the event of clinical dysentery, empiric antimicrobial therapy is routinely given by the clinics.

### **6.6 Future provision of micronutrient supplements**

Multivitamins are not routinely provided for children in the District. It is not intended to provide children who complete the study with multivitamins/micronutrients. Once the data has been analyzed, and if a significant benefit is observed, then we will work with the District, Provincial and National health authorities, with whom we have close working relationships, to recommend the optimal intervention as a standard of care practice.

### **6.7 Toxicity Management**

There are no reported side effects of vitamins/micronutrients in the doses proposed in this study, which are essentially 1 RDA. A number of studies, and practice in some areas, have used multiples of the RDAs for some of the micronutrients in daily doses, and have not noted toxicity. Children will be closely followed for morbidity with weekly visits. Specific questions will be asked about respiratory and GI symptoms, along with more open-ended questions about other symptoms. Once the code is broken we will have a sense of whether there was any toxicity (or benefit) associated with one of the three regimens. Because there are no known toxicities of the micronutrients in the doses to be used, it is hard to anticipate any toxicity.

If a child has a severe illness that does not have an identifiable cause, we will leave it open to the clinician caring for the child as to whether or not to ask the study team to break the code on that child. Most children who become severely ill are cared for in the District Hospital which is situated in our study area. The physicians working at the hospital will thus be familiar with this study. We will give parents of children participating in the study a plastic folder with a card with their study details which they will keep, and can present when going for care.

In the event of an overdose of the study treatment, the treatment would be stopped and the study physician would see the infant weekly for 2 weeks and note any side effect. The treatment schedule would be discussed and clarified again with the parent/guardian and the infant restarted on treatment.

Any child having a moderate or severe adverse event that is probably related to micronutrient supplementation will be monitored weekly for 4 weeks (or longer as necessary). Micronutrient supplementation will be discontinued until the relationship to the adverse event can be established. If the adverse event is not thought related to micronutrient supplementation, the supplementation will be re -instituted.

Such events will come to the attention of the staff in one of two ways. One is from visits by the field workers to home; the other is by visits to local clinics or hospitals. The field staff will report directly back to the medical staff on the study. For clinic staff we will ask them to call a physician member of the study team, one of whom will always be carrying a mobile phone for the study. The number for the mobile phone will be on the study card given to patients. In this way we hope that any adverse events will be evaluated within a few hours of their coming to the attention of a study staff member or a medical care provider at a clinic or hospital.

For definitions of moderate or severe, or possible and probable (in relation to adverse event) please see Appendix III. Grade 3 and Grade 4 skin reactions in this context would be considered serious adverse reactions and treatment stopped if they were considered possibly or probably related to micronutrient supplementation.

### **6.8 Criteria for Treatment Discontinuation**

6.81 Subjects who complete treatment as defined by the protocol

- 1. Moving out of Hlabisa District
  2. A moderate or severe adverse event possibly or probably related to the micronutrient intervention

6.83 Death

6.84 Parents/Guardians withdrawing consent

6.85 Subject/Physician requests discontinuation of study treatment. If the parent/guardian requests that the infant should receive no further medication then we would request that home and clinic visits to document morbidity would continue. In this situation the infant would be included in the intention to treat analysis. If the parent/guardian requests that the home visits should cease then we would respect that decision and the infant would be removed from the study and data analyzed only for the time that the patient was in the study.

6.86 Subjects who are non-adherent with nutritional supplements and/or clinic visits would remain in the study as long as we continue to have home monitoring of morbidity events.

## 7.0 Statistical considerations

### **7.1 General Design Issues**

For the purpose of sample size calculation the primary outcome of this study is to compare the efficacy of two micronutrient supplements – multiple micronutrients including zinc and vitamin A, versus zinc and vitamin A - to vitamin A in three different groups of children; HIV-infected children, HIV-uninfected children born to HIV-infected mothers, and HIV-uninfected children born to mothers who were not HIV-infected to reduce the prevalent days of diarrhea. Primary exposure variables are HIV infection status of the child, HIV infection status of the mother, and treatment. Potential confounding variables include sex of child, age, parity, nutritional status of the mother, nutritional status of child on entry to the study, vitamin A and zinc status on entry into the study, breast-feeding pattern, water use pattern, and socioeconomic status of the family. For HIV-infected children CD4 counts, viral load and clinical stage of HIV infection (using CDC criteria) will also be examined as potential confounding variables.

Because the infants who are HIV-uninfected at enrollment may later become infected if they are exposed to breastmilk or other risk factors, the HIV status of children will be treated as a time dependent variable.

The primary statistical comparisons to be made are:

1. Vitamin A versus zinc and vitamin A supplementation;
2. Vitamin A versus zinc, vitamin A plus micronutrient supplementation;
3. Zinc and vitamin A supplementation versus zinc and vitamin A plus micronutrient supplementation;

for each of the three groups of children.

### **7.2 Endpoints**

7.21 Primary Endpoints

The primary outcome measure is prevalent days of diarrhea per child during the 18 months that children will receive supplements (from 6 to 24 months of age). Comparisons will be stratified by HIV status, and all three micronutrient regimens will be compared within these strata. “**Prevalent days of diarrhea will be assessed as follows. The information on diarrhoea is collected during weekly home visits. The procedure is as follows.(1) If the mother answered ‘YES’ to the question whether the child had any diarrhoea since the last home visit, and (2) If the mother answered ‘YES’ to one or more of the questions relating to presence of stools looser than normal, more frequent than normal or stools with blood. If these two conditions are fulfilled then the mother is specifically asked, for every weekday since last visit, whether the unusual stools were present on that day. This process is repeated weekly and recorded on the field monitoring forms. Prevalent days of diarrhoea will be expressed as the total number of days of diarrhoea over the total number of days of follow-up for each child.**”

- 1. Secondary Endpoints
     1. Pathogen-specific pattern of enteric infections in both HIV-infected and uninfected children, by treatment arm, between 6 and 24 months.
     2. Total cost of illness during 18 months of supplementation, compared between treatment arms in each cohort.
  2. Tertiary Endpoints
     1. a) Linear growth and body composition in different intervention arms
     2. Pathogens associated with the development of persistent diarrhea lasting >14 days.

### **7.3 Sample Size and Accrual**

**Expected treatment effects on diarrhea prevalence by HIV status**

*(Note. There will not be a group who receive no intervention. The columns below are included to permit comparison with other published studies in which placebos were used.)*

|  | Expected days of diarrhea during 18 m study period **No intervention** | Expected days of diarrhea in control arm **Vitamin A** | Expected days of diarrhea in intervention arm 1 **Zinc only** | % Treatment effect compared with the control arm | Expected days of diarrhea in intervention  arm 2 **MVS + Zinc** | % Treatment effect compared with the control arm |
| --- | --- | --- | --- | --- | --- | --- |
| HIV-infected | 27± 6 | 24± 5 | 20± 5 | 17% | 16± 5 | 33% |
| HIV-uninfected | 20± 5 | 18± 5 | 15± 5 | 17% | 12± 5 | 33% |

For sample size calculation we made the following assumptions. The expected mean days of diarrhea (± standard deviation) during the 18 months of study for the HIV-infected children given vitamin A is 24 ± 5 days. For uninfected children given vitamin A it is 18 ± 5 days. Sample size calculations are based on being able to detect 17% differences between the vitamin A and zinc supplemented groups, and being able to detect 20% differences between the zinc and the micronutrient and zinc groups. These detectable differences require a sample size of 26 children for each arm of the HIV-infected groups and 45 for each arm of the HIV-uninfected groups (significance level of 0.05 and a power of 0.8 and based on two-sided two sample t-tests).

It is expected that mortality, migration, and dropouts during the study in the HIV-uninfected groups will be no more than 25%. The mortality within the HIV-infected group is likely to be significantly higher and we have assumed a 50% attrition rate within this group during the 18 month study period. Thus the sample size requirements for each arm are increased from 26 to 52 in the HIV-infected group, and from 45 to 60 in each arm of the HIV-uninfected children. Thus the total initial enrollment required will be 156 HIV-infected children and 360 children who are not HIV-infected, with the final sample size being 78 HIV-infected children (26 in each treatment group after 50% attrition), and 270 uninfected children (45 in each treatment group after 25% attrition). Data from all children who die or dropout from study will be analyzed for the period that they were in the study. The number of HIV-infected children born among mothers recruited to the VTS will be approximately 70 per year. If we assume that 80% of eligible HIV-infected children will be enrolled in the study, recruitment will take approximately 36 months. We will consider making the recruitment more rapid by expanding to clinics outside the VTS but keeping all other procedures in place. If we do so we will notify the NIH.

### **7.4 Monitoring and Analysis**

7.41 Analysis plan

Data for the primary and secondary endpoints will be analyzed by intention to treat. Data will be examined graphically for normality assumptions and summarized by standard statistics. The principle hypotheses will be tested by t-tests if normality can be assumed and by the Mann-Whitney test if not. Missing data involved with our primary endpoint, prevalent days of diarrhea, will necessitate certain assumptions for those individuals who drop out. These assumptions (worst case or predicted) will also be explored, but we have powered the study to adjust for dropout rates so that we can analyze it as an efficacy study. The sample size also allows for comparisons within each stratum of mother: infant HIV status as well as between strata.

We will take advantage of the factorial design to understand the benefits of micronutrients and their possible limitations according to host factors. While our design plan and sample size calculations were based on selecting certain specific linear contrasts other outcome variables, inter-relationships among the outcome variables and effects of potential confounding fit into a factorial ANOVA design and will be explored using ANOVA methods as well as multiple regression analyses of continuous covariates.

Assigning children who are initially HIV-uninfected and become HIV-infected during the course of the study will take a considered approach. Because the study will determine HIV status every 3 months, if status is only reassigned at the time that the test is positive the duration of HIV infection will be underestimated.

We will adopt two approaches to control for this information in the analysis:

1. The primary outcome data will be collected and recorded along with HIV status for pre-specified intervals – that is every 3 months. This data will be analyzed longitudinally using generalized estimating equation (GEE) methods, with measurements made at multiple time points for each child. For each child at each time point, the outcome or dependent variable will be the number of days of diarrhea during the past 3 months. Independent variables would include current HIV infection status of child, HIV infection status of mother, treatment arm and other covariates such infant CD4 counts.
2. HIV infection status of child at end of study, HIV infection status of mother, length of time child was infected with HIV (mid-point between first positive result and last negative result), treatment arm and other covariates including infant CD4 counts will be included as covariates in a regression model.

7.42 Interim analyses

Two interim analyses of efficacy data will be performed:

1. When half the study population has completed 6 months of follow-up, and,
2. When half the study population has completed 18 months of follow-up.

The sample size as it stands is sufficient to permit these two interim analyses and the final analysis with 80% power for each of the comparisons.

These results will be forwarded to the DSMB. In addition, the study team on site will review 3 monthly assessments of recruitment and follow-up rates and treatment adherence rates.

Data will be gathered and entered in to a SQL database and summarized and analyzed using SPSS.

## 8.0 Human subjects

### **8.1 Institutional Review Board (IRB) Review and Informed Consent Process**

This protocol and the informed consent document (Appendix II) and any subsequent modifications will be reviewed and approved by the Ethics Committee of the Nelson R. Mandela School of Medicine, University of Natal, Durban, which is responsible for oversight of the study. The study protocol will also be submitted to the Institutional Review Board of New England Medical Center/Tufts University School of Medicine. Written informed consent will be obtained from parent or legal guardian. The informed consent document describes the purpose of the study, the procedures to be followed, and the risks and benefits of participation. A copy of the consent form will be given to the parent or legal guardian.

### **8.2 Subject Confidentiality**

Fieldworkers need to visit children at home. Study forms for use in household visits will contain the child and family name. A study number will also be recorded on this sheet. Initial data verification will use this external study number. This external study number will be linked to an internal study number. All subsequent data manipulation and access will utilize the internal study number. The internal study number that will allow linking of identifiers with HIV status will only be accessible to senior study scientists. In this way personal details will be protected. In particular, HIV results, identified only by a lab bar code number, will be linked to the internal study number and will never be generally available to centre staff or fieldworkers.

All records will be kept at the headquarters of the Africa Centre in a secure place. All computer entry and networking programs will be done with coded numbers only. HIV infection status of mothers and children will be protected information. Clinical information will not be released without written permission of the subject, except as necessary for monitoring.

### **8.3 Study Discontinuation**

The study may be discontinued at any time by the NIAID or NICHD.

## 9.0 Plans for distribution of research findings to study community

The Africa Centre works closely with the local, provincial, and national governments to disseminate the findings of the studies it conducts. In addition to these links with government, the Centre operates a Community Liaison Office, and a Community Advisory Board (CAB, Appendix IV). The CAB meets monthly, and the results of this study will be presented at that meeting. Before the study is initiated, it will also be discussed in detail with the CAB. Should one or more of the micronutrient interventions prove effective in this study, we would discuss with the above mentioned stakeholders the import of our findings, and provide recommendations on how they should be utilized. Because South Africa has a strong community health infrastructure, decisions about implementation are made by the government, and not NGOs acting independently.

If one of the micronutrient interventions evaluated in this study is effective in the age group being studied (6 –24 months) that does not mean that the intervention will also be effective in older children. We would not therefore lobby for, or supply, micronutrients to children when they finish this study at age 24 months. We would however consider additional research to evaluate the efficacy of these micronutrient interventions in older children.

## 10.0 Biohazard containment

Because transmission of HIV and other blood-borne pathogens can occur through contact with contaminated needles, blood, and blood products, appropriate blood and secretion precautions will be employed by all personnel in the drawing of blood and shipping and handling of all specimens for this study, as currently recommended by the U.S. Centers for Disease Control and Prevention. All staff who take blood or who may be in contact with potentially infected materials will receive specific training from the Africa Centre, which will be repeated every 12 months. Similarly all staff taking blood or who may be in contact with potentially infected materials are required to be tested for hepatitis B and where indicated receive immunizations.

The Africa Centre has a policy in place on occupational exposure to infectious agents, and prevention if there is exposure. This policy will be followed for this study.

### **10.1 Specimen handling and shipping**

Stool samples collected in the field will be labeled and transported from the field to the study office in portable cool boxes similar to ones routinely used for transporting vaccines. Rectal-swab specimens will be put in BGS media for transport for culture, and into PBS for viral ELISA determination. Stool samples will be transported on the same day of collection to Durban where they will be inoculated onto the appropriate plates and incubated overnight. When transport is not available on the same day, stool samples for bacterial culture will be inoculated at the Africa Centre field laboratory, while samples for protozoan and parasitic examination and for viral studies will be refrigerated prior to transport the following day.

Samples collected in the field will be logged at the study office and also at sample repository in Durban. A comprehensive archiving programme is used in the sample repository to log receipt, testing and residual sample volume for further analysis.

A bus installed with both a refrigerator and a freezer travels every working day between the Africa Centre and the Africa Centre Durban laboratories. The longest time between sample collection and delivery to the laboratories in Durban should not be more than 10 hours.

A field laboratory has been established at KwaMsane, one of the study clinics. Basic procedures such as microscopy and centrifugation can be performed here by laboratory staff .

All infectious specimens to be shipped outside the Centre will be sent using the ISS-1 SAF-T-PAK mandated by the International Air Transport Association Dangerous Goods Regulations-Packing Instruction 602. Please refer to individual carrier guidelines, e.g., FedEx, Airborne, for specific instructions.

## 11.0 References

1. Berhens R, Lunn P, Northrop C. Factors affecting the integrity of the intestinal mucosa of Gambian children. Am J Clin Nutr. 1987;45:1433-1441.
2. Roy S, Tomkins A. The impact of experimental zinc deficiency on growth, morbidity and ultrastructural development of intestinal tissue. Bangladesh Journal of Nutrition. 1989;2:1-7.
3. Tomkins A, Behrens R, Roy S. Micronutrient supplements for diarrheal disease. Hong Kong Journal of Pediatrics. 1995;suppl:95-99.
4. Sachdev H, Mittal N, Yadav H. A controlled trial on the utility of oral zinc supplementation in acute dehydrating in infants. J Ped Gastroenterol Nutr. 1988;7.
5. Sazawal S, Black R, Bhan M, Bhandari N, Sinha A, Jalla S. Zinc supplementation in young children with acute in India. New England Journal of Medicine. 1995;333:839-844.
6. Roy S. Zinc supplementation in the treatment of childhood diarhoea. Indian J Pediatr. 1995;62:181-193.
7. Sazawal S, Black R, Bhan M, et al. Oral zinc supplementation in persistent diarrhea in infants. Ann Trop Pediatr. 1990;10.
8. Bhandari N, Bhan M, Sazawal S. Mortality associated with acute watery , dysentery, and persistent in rural north India. Acta Pediatrica Scand. 1992;381.
9. Ninh NX, Thissen JP, Collette L, Gerard G, Khoi HH, Ketelslegers JM. Zinc supplementation increases growth and circulating insulin-like growth factor I (IGF-I) in growth-retarded Vietnamese children. Am J Clin Nutr. 1996;63:514-519.
10. Rosado JL, Lopez P, Munoz E, Martinez H, Allen LH. Zinc supplementation reduced morbidity, but neither zinc nor iron supplementation affected growth or body composition of Mexican preschoolers. Am J Clin Nutr. 1997;65:13-19.
11. Thea D, St Louis M, Atido U, et al. A prospective study of and HIV-1 infection among 429 Zairian infants. New England Journal of Medicine. 1993;329:1696-1702.
12. Powell K. Approach to gastrointestinal manifestations in infants and children with HIV infection. J Pediatr. 1991;119:S34-S40.
13. Smith P, Lane H, Gill V. Intestinal infections in patients with the acquired immunodeficiency syndrome (AIDS). Ann Intern Med. 1988;108:328-333.
14. Oshitani H, Kasolo F, Mpabalwani M, et al. Association of rotavirus and human immunodefiency virus infection in children hospitalized with acute , Lusaka, Zambia. Journal of Infectious Diseases. 1994;169:897-900.
15. The Italian Pediatric HIV Study Group. Intestinal malabsorption of HIV-infected children: relationship to , failure to thrive, enteric microorganisms and immune impairment. AIDS. 1993;7:1435-1440.
16. Mayer H, Wanke C. Diagnostic strategies in HIV-infected patients with . AIDS. 1994;8:1639-1648.
17. Rollins NC, Kindra G et al. CD4 count, HIV viral load and enteric pathogens in HIV-Infected children with persistent in Durban, South Africa. Abstract 750931. Pediatric Academic Societies & American Academy of Pediatrics Joint Meeting, Boston 12-16^th^ May 2000.
18. Coutsoudis A, Bobat RA, Coovadia HM, Kuhn L, Tsai WY, Stein ZA. The effects of vitamin A supplementation on the morbidity of children born to HIV-Infected women. Am J Publ Health 1995;85: 1076-1081.
19. Willumsen J, Darling J, Kitundu J, et al. Dietary management of acute in children: effects of fermented and amylase-digested weaning foods on intestinal permeability. Journal of Pediatric Gastroenterology and Nutrition. 1997 Mar;24(3):235-41.
20. Widmer G, Tzipori S, Fichtenbaum CJ, Griffiths JK. Genotypic and phenotypic characterization of Cryptosporidium parvum isolates from people with AIDS. J Infect Dis. 1998;178:834-840.
21. Carraway M, Tzipori S, Widmer G. Identification of genetic heterogeneity in the Cryptosporidium parvum ribosomal repeat. Appl Environ Microbiol. 1996;62:712-716.
22. Carraway M, Tzipori S, Widmer G. A new restriction fragment length polymorphism from Cryptosporidium parvum identifies genetically heterogeneous parasite populations and genotypic changes following transmission from bovine to human hosts. Infect Immun. 1997;65:3958-3960.
23. Vasquez JR, Gooze L, Kim K, Gut J, Petersen C, Nelson RG. Potential antifolate resistance determinants and genotypic variation in the bifunctional dihydrofolate reductase-thymidylate synthase gene from human and bovine isolates of Cryptosporidium parvum. Mol Biochem Parasitol. 1996;79:153-165.

# Appendices

## Appendix I Schedule of Events

**Schedule of tests and Assessments**

|  | 4 mths | 6 mths | 9 mths | 12 mths | 15 mths | 18 mths | 21 mths | 24 mths | diarrheal event |
| --- | --- | --- | --- | --- | --- | --- | --- | --- | --- |
| Hemoglobin |  | X | X | X | X | X | X | X |  |
| Serum zinc |  | X |  | X |  | X |  |  |  |
| Serum retinol |  | X |  | X |  | X |  |  |  |
| Serum tocopherol |  | X |  | X |  | X |  |  |  |
| Serum RBP |  | X |  | X |  | X |  |  |  |
| C-reactive protein |  | X |  | X |  | X |  |  |  |
| Plasma HIV RNA | • | • | X* | X* | X* | X* | X* | X* |  |
| CD4 count |  | • |  | X* |  | X* |  | X* |  |
| Stool analysis |  | 🟑 |  | 🟑 |  | 🟑 |  | 🟑 | X |
| Anthropometry |  | X | X | X | X | X | X | X |  |
| Body composition |  | X |  | X |  | X |  | X |  |

Note: Plasma HIV RNA measurement at 4 months will be performed as part of the Vertical Transmission Study and not as part of this protocol. The test is reflected, however, in this table because it determines the HIV status of infants which is a pre-requisite for entry into this study.

• Qualitative and quantitative HIV RNA and CD4 counts

X* Quantitative HIV RNA testing

🟑 Stool analysis

◆ Only HIV-infected children to have a lactulose: mannitol test and urinary creatinine at age 12 month

# APPENDIX II: INFORMED CONSENT FORM

**AFRICA CENTRE FOR HEALTH AND POPULATION STUDIES**

**and**

**TUFTS-NEW ENGLAND MEDICAL CENTER**

## INFORMATION SHEET AND CONSENT FORM FOR PARTICIPATION

**MICRONUTRIENTS AND ENTERIC INFECTION IN AFRICAN CHILDREN**

We are inviting you and your child to participate in a research study being conducted by the Africa Centre.

**What do we want to find out?**

In this research study we want to find out if children given vitamins and minerals can be protected from diarrhea caused by germs. We are investigating which vitamins and minerals can improve a child’s weight and growth. We also want to find out if the same thing happens in children who do or do not have HIV, the virus that causes AIDS.

This is important as many children in South Africa and other developing countries get these diseases. They also do not grow adequately. This research study will help us find out if these vitamins and minerals, which are inexpensive, can help children in South Africa. A total of 516 children will participate in this research study.

**What are these vitamins and minerals?**

In this research study we will compare three types of mixtures of vitamins and minerals to find out which works better. One of the mixtures will have vitamin A alone. Vitamin A is used internationally in trying to improve the health of children. The government of South Africa has recommended that it be used in children. The second mixture will include vitamin A and zinc. Zinc has been shown to improve the health of children in Asia. We do not know if it will work in children in Africa. The third mixture will include vitamin A, zinc and other vitamins and minerals including vitamins C, D, E, K, B^1^, B^2^, B^6^, B^12^, iron, niacin, folate, iodine and copper.

.

**What do we request you to do?**

If you agree that your child participates in this research study, we will give him/her one of these mixtures daily for eighteen months. We will request that you give this to your child by mouth. These vitamins and minerals will be in tablet form, and can be crushed in water or in food like porridge. Your child must be able to eat and drink to be able to do this. We will take blood samples seven times in the eighteen-month period. Four times we will use a needle and syringe. Every time we take blood the amount will be the size of a teaspoon and a half. We will take blood again three times by pricking the child’s finger or heel. Whenever we do this, we will only take a few drops of blood.

We will also take a stool sample from your child when the child comes to the clinic.

As part of the study, we will also request that you bring your child to the clinic every three months. At the clinic, we will weigh the child. We will also measure the amount of fat and flesh using a machine. This is not painful. There is a picture attached to this form showing how this machine works in an adult.

**Where and how will we see you?**

One member of the study team will visit you once weekly. This person will bring the vitamin mixture and watch while you give it to you child. We will give your child the vitamin mixtures and continue to visit you until the child is two years old.

We will also ask that you keep a diary to record daily when the child has diarrhoea. If your child has diarrhoea, we will request that you take a stool sample for us. We will test this sample to find out what the cause of the diarrhoea was.

**What kinds of questions will be asked?**

During the clinic visits and during the home visit, you will be asked questions about diarrhoea and other diseases and their treatment as well as the costs of that treatment.

If the study finds that the vitamins can prevent diarrhea we need to know if this is a treatment that South Africa can afford for its children. To know this we need to learn what the costs are for the families of children who get sick with diarrhea and other illnesses. Illness in a child can cost families in several ways: it can cost money to buy medicines, money to see a traditional healer, and money to travel to a clinic or hospital. It can also cause families to lose money because parents cannot go to work, as they have to care for the child or bring the child to the clinic. We also want to try and learn how illness in other family members, such as a parent, can affect a family’s ability to take care of children in the family.

We will look at the records of any children who are admitted to the district hospital or seen in the clinics when they are sick and add up the costs caused by such visits. The field workers will ask you questions each week when they visit if your child has been sick in the preceding week. These questions will be about the costs you have to pay due to any illness in your child or other family members. You will be asked about the cost of any medicines you may have bough, the costs of any services you received from doctors or traditional healers, the cost of travel to clinics and hospitals, the cost of any meals and lodging you had to pay for at the hospital, and the loss of any work wages due to having to care for the child or due to personal other family member illness. You will also be asked about the effect the illness has had on your family in terms of missed work, missed schooldays by siblings, and reductions in buying food or other household items due to the spending of money on illness related costs”

**Which vitamins and minerals will your child receive?**

At the time of the study, no member of the study team will know the type of vitamin tablet received by your child. We will not know this until the study is complete. The decision which type of tablet your child gets will be by chance. This is like flipping a coin in deciding which child is assigned to get which vitamins and minerals. But all tablets will look and taste the same. If you were planning to give your child additional vitamins, do not enroll in the study.

**Will these vitamins help or harm your child?**

If your child participates in this study, he/she may have fewer days suffering from diarrhoea than other children. These tablets may also help with your child’s growth. It may also happen that these tablets do not help your child in any way.

There are no reasons to think that these tablets will harm your child in any way. The taking of blood may be a little painful, but this will cease after a minute or two. There may be a bruise where the blood was taken.

**Are there any costs or financial compensations involved?**

There will be no costs involved. The vitamin and mineral tablets will be given to you free of charge.

### ***How will confidentiality be maintained?***

All the information that the research study will collect on you and your child will be treated as strictly confidential. It will be kept anonymously in a secure place and nobody will ever be given information on either your participation, your health or your child’s health, any test results or measurement results or any of your answers to our questions or other personal information.

**What happens when you cannot decide whether to participate or not?**

The decision to participate or not is entirely yours. You need not decide now – you may consult your family and delay your decision. If you prefer not to participate in the study, feel free to say “no”. If you are not happy about the way things are going at a later time, you may withdraw without any problems. If your child is in this study, he/she may not enroll in another study.

**What happens when you decide not to participate or to stop participation?**

Not participating with this research study is a valid alternative. If you prefer not to participate or to stop participation, this will not affect the quality of the care your child will receive: you can still visit the clinic and receive care like before.

**How long will the study last? How many mothers and children will take part?**

This study will take approximately four years in the Hlabisa district. The study period may be shorter if the vitamins work well. When the study is complete, we will inform you about the results. We will collaborate with district and provincial health managers, and inform them about the results of the study. We intend to enroll five hundred and sixteen mothers and children in this study.

**Who can you contact for further information?**

If you need additional information before making your decision whether to participate or not, or when already enrolled and requiring more information, call any of the following doctors at the Africa Centre:

Dr.Michael Bennish

Tel: 035.5507502

Dr.Van den Broeck

Tel: 035.5507546

Tel: 083.5186945

We will give you a booklet with rights of study participants. Whenever you have questions about rights of study participants, please contact the Community Liaison Office of the Africa Centre:

Mrs. Dudu Biyela, Community Liaison Office

Africa Centre, Mtubatuba

Tel: 025 550 7514

Or phone the Tufts-New England Medical Centre Institutional Review Board

At: 09.1.617.636-7512 This latter number is in the United States and you will have to pay the costs of the call to reach them. Also, the time is different in the United States than it is in South Africa. Offices in the United States will only open when it is about 15:30 hours here (3:30 PM).

**PARTICIPANT’S STATEMENT**

**Consent for Study “Micronutrients and Enteric Infection in African Children”**

**Africa Centre for Health and Population Studies**

I have been informed by ______________________about the purpose of the study, its procedures, risks and benefits. I have been informed what the investigators expect of me. I have been given the opportunity to ask questions which have been answered to my satisfaction.

I know that participation is voluntary and that I may withdraw from the study at any point and that, if I do so, this will have no effect on my child’s or my own future health care at the clinic.

I know that if I have any question concerning my right as a participant, I may contact Mrs.Dudu Biyela at the Africa Centre, whose phone number is 035.5507514. I may also contact the Tufts-New England Medical Centre Institutional Review Board in the United States at: 09.1.617.636-7512

I understand that as a participant in this study my identity and my medical records and data relating to this research study will be kept confidential.

I agree that my child, _______________________________________________________

participate in the study “Micronutrients and Enteric Infection in African Children” being conducted by the Africa Centre. I agree with the study procedures as they have been described to me. I agree that a trained researcher may review my child’s medical records should my child be admitted to hospital or seen in the clinic during this study.

Parent/ Guardian’s Signature:

__________________________________ Date _____________________

Representative of Principal Investigator’s Signature:

__________________________________ Date _____________________

Witness’s Signature:

__________________________________ Date _____________________

You may keep one of these forms, the other will be kept at the Africa Centre

## Appendix III Adverse Events

**Reporting events**

Field workers will report difficulties encountered at the home on a ‘*Difficulty report form’*. The term ‘difficulty’ is used to facilitate consistency with terms used by the breastfeeding counselors in the Vertical Transmission Study (VTS). The WHO breastfeeding counseling course discourages the use of “problem” when counseling a mother who is breastfeeding her child. The use of “difficulty” is preferred. Field staff in the VTS will therefore report ‘difficulties’ to their supervisors. Supervisors, in turn, will visit the home and document in greater detail whether an adverse event has occurred. This shall be reported on an ‘*adverse event form’*. If a child is admitted to hospital or dies, then the project nurse will visit the hospital and/or home to collect details of the event and record these on the adverse event form.

The same system will be used in this study.

Only supervisors or project nurse/physician will be allowed to complete the *adverse event form*. Once returned to the study office all *adverse event forms* will be reviewed by the study physician before entry into the database.

By using a smaller group of highly trained staff to verify and confirm adverse events we aim to improve the accuracy of reporting and understanding any relationship to the interventions.

**Relationship of adverse event to micronutrient supplements**

An adverse event is defined as any undesirable experience occurring to a patient during the study whether or not related to the study drug. A serious adverse event is defined as one that is life threatening, fatal, disabling or results in prolonged hospitalization or readmission. Adverse events will be assessed for their relationship to the study drug and classified as none, remote, possible, probable or not assessable. The assessment of the relationship of an adverse event to the administration of study drug (none, unlikely (remote), possible, probable, not assessable) is a clinical decision based on all available information at the time of the completion of the case report form.

**None** - includes: (1) the existence of a clear alternative explanation (e.g. mechanical bleeding at surgical site); or (2) non-plausibility (e.g., the patient is struck by an automobile at least where there is no indication that the drug caused disorientation that may have led to the event; cancer developing a few days after drug administration).

**Unlikely (remote)** - a clinical event, including lab abnormality, with an improbable time sequence to drug administration and in which other drugs, chemicals or underlying disease provide plausible explanation.

**Possible** - a clinical event, including laboratory abnormality, with a reasonable time sequence to administration of the drug, which could also be explained by concurrent disease* or other drugs or chemicals. Information on drug withdrawal may be lacking or unclear.

**Probable** - a clinical event including lab abnormality, with a reasonable time sequence to administration of the drug, unlikely to be attributed to concurrent disease* or other drugs or chemicals, and which follows a clinically reasonable response on withdrawal (dechallenge).

**Not assessable** - a report of an AE which cannot be judged because information is insufficient or contradictory, and which cannot be supplemented or verified.

* Concurrent disease includes concomitant, intercurrent and underlying disease/condition. Concomitant disease - any other illness the subject may have at the time of entering the clinical trial. Intercurrent disease - any other illness the subject may develop during the clinical trial. Underlying disease - the illness which is the indication for study drug therapy.

Factors to be considered include:

• **The temporal sequence from drug administration** (The event should occur after the drug is given. The length of time from drug exposure to event should be evaluated in the clinical context of the event.)

• **Recovery on discontinuation (dechallenge), recurrence on reintroduction (rechallenge)** (Subject’s response after drug discontinuation (dechallenge) or subjects response after drug re-introduction (rechallenge) should be considered in the view of the usual clinical course of the event in question.)

• **Underlying, concomitant, intercurrent diseases** (Each report should be evaluated in the context of the natural history and course of the disease being treated and any other disease the subject may have.)

• **Concomitant medication or treatment** (The other drugs the subject is taking or the treatment the subject receives should be examined to determine whether any of them may be recognized to cause the event in question.)

• **Known response pattern for this class of drug** (Clinical/preclinical.)

• **Exposure to physical and/or mental stresses** (The exposure to stress might induce adverse changes in the recipient and provide a logical and better explanation for the event.)

• **The pharmacology and pharmacokinetics of the test drug** (The pharmacokinetic properties (absorption, distribution, metabolism and excretion) of the test drug (s) the subject is taking, coupled with the individual subject 's pharmacodynamics should be considered.)

**Intensity (severity) of the event**

The following classification should be used:

**Mild:** usually transient in nature and generally not interfering with normal activities.

**Moderate:** sufficiently discomforting to interfere with normal activities.

**Severe:** prevents normal activities.

## Appendix IV Community Advisory Boards

Two community advisory boards (CAB) have been established in the area. The HIV Vaccine Preparedness Research Group established the first of these, and serves the Hlabisa Tribal Area of the Hlabisa District. The second serves the Mpukunyoni tribal area of the Hlabisa District and works closely with the Africa Centre.

The Mpukunyoni CAB consists of 33 members and meets monthly. The tribal area is divided into separate ‘isigodis’ or wards. There is one elected representative from each ward. In addition, there is one representative from each of the main government sectors, namely Agriculture, Education, Health and Welfare and also one representative from the Traditional Healers Association.

The Mpukunyoni CAB has a constitution with stated mission and objectives.

**Objectives**

- Establish a sound relationship between Africa Centre and the community.
- To act in an advisory capacity to the community and Africa Centre.
- To keep the community informed of all research that takes place in their area.
- To discuss, evaluate and recommend questionnaires before they are taken to the community.
- Bringing to researcher’s attention anything that has any negative impact on the community.
- To advice Africa Centre of the correct protocol in the researched community.

The Africa Centre Community Liaison Office is also working with the CAB to identify other local priorities and develop response plans. Together, they are about to distribute their first newsletter highlighting the CAB activities and those of the Africa Centre. The Community Liaison Office of the Africa Centre has provided training on ethics and has also sponsored individuals to attend courses on research ethics. Members of the CAB represent the community on the University of Natal, Durban Ethics Review Committee.

## Appendix V Laboratory Methods

**Detection of Enteric Pathogens**

Stool samples will be examined for *Campylobacter*, *Salmonella*, and *Shigella* using standard methods. Briefly, the study laboratory assistant at the Africa Centre field laboratory will inoculate stool onto MacConkey and XLD agars and into selenite broth. These plates and broth will then be transported to the microbiology laboratories of the University of Natal Medical School. The selenite broth will be sub-cultured onto MacConkey agar after 24 hours of incubation at 42^0^C. Suspected colonies will be screened for urease production and if negative further identified by means of standard biochemical and agglutination tests. *Campylobacter* *jejuni* will be grown on Butzler’s medium and identified by standard methods. *Vibrio cholerae* will be searched for only if clinical cholera is present in the community (as it currently is). We will not attempt to culture *Mycobacterium* *avium-intracellulare.*

Ten lactose-fermenting colonies with morphology typical of *Escherichia coli* will be picked from the McConkey plate, grown in TSB culture, and frozen at -70° C in TSB-15% glycerol. If funds are available, these isolates will subsequently be probed for genes coding for virulence factors of diarrheagenic *E. coli*. This will be done by means of DNA probes for the LT and ST genes (human) for ETEC, EAF and EA factor genes for EPEC, invasin gene for EIEC, AA gene for EaggEC and the DA gene for DAEC. Because EHEC has proven to be uncommon in developing countries, we will probe for SLT-I and SLT-II genes only in children with bloody diarrhea who do not have *Shigella* isolated from their stool. Probes for genes will be produced in Durban using standard methodology. The presence of the genes will be established by means of DOT-blot technique.

Rotavirus, enteric adenoviruses 40/41, astroviruses and Norwalk virus will be detected by ELISA with reagents supplied by Dr. Steele of Medunsa Medical School. Stool specimens for viral detection will be frozen after arriving in the Durban laboratories and tested in batch.

Protozoan pathogens: A major focus of this study will be on the detection of protozoan pathogens. Collection of specimens, storage, and microscopic identification of these agents will be done as follows:

Stool specimens will be examined fresh as soon as is feasible after arrival in Durban and aliquots will also be stored preserved in 10% buffered formalin at 4°C. *Entamoeba histolytica* and *Giardia lamblia* will be detected initially by wet eosin mount. A portion of the fresh stool will be concentrated for better detection of oocysts. Liquid stools will be centrifuged at 500 x g for 10 minutes, the supernatant decanted, and the sediment subjected to formal saline ether sedimentation, followed by saturated sodium chloride flotation for detection of *C. parvum* oocysts. Slides will be prepared using 10 ul aliquots of the resulting sediment. One slide will be stained with a modified acid fast stain, and the other slide stained with a modified trichrome (chromotrope 2R) stain. Protozoan pathogens will then be detected by their characteristic morphology:

- *Cryptosporidium*: round, 4-6µm, bright red oocysts with background of blue green fecal debris and yeast on the acid-fast stain.

*- Cyclospora*: round, 8-10µm, pink/red/purple against blue green background on the acid-fast stain.

*- Isospora*: elliptical, 20-30µm by 10-20µm, bright red oocysts with blue-green background on the acid-fast stain.

*- Microsporidia* spores: 0.5-1.5µm, bright pink with a central stripe on trichrome stain. Spores of *Enterocytozoon bieneusi* are small (0,5 x 1,5 um) and oval in shape while those of *Encephalitozoon intestinalis* are larger (1.8 x 1.00 m).

#### **Immunofluorescence**

A commercially available kit made by TechLab will be also used for detecting *C. parvum* and *G. lamblia* by immunofluorescence.

#### **Genotyping of Cryptosporidium parvum**

Human isolates of *C. parvum* have been classified into two genetically distinct genotypes, genotype I (human-type) and genotype II (bovine-type). The genotypes of *C. parvum* isolated from children with and without HIV infection will be analyzed and compared. A subset of 150 fecal samples (75 from HIV-infected and 75 from non HIV-infected children) positive for *C. parvum* by microscopy will be analyzed for isolate genotype. Fecal samples will be stored in 2.5% potassium dichromate at 4C till analysis. Oocysts will be purified by salt flotation and Percoll density gradient centrifugation.(20) DNA will be extracted from fecal samples and genotyped by RFLP analysis of PCR-derived DNA fragments.(20) Analysis will be restricted to three previously identified and published polymorphic loci, namely ITS1 region of rDNA,(21) threonine-rich ORF,(22) and dihydrofolate reductase-thymidilate synthase (DHFR-TS) gene.(23) The genotype of isolates from *C. parvum*-infected HIV-positive and negative individuals will be compared and correlated with epidemiological and clinical data

***Assays to Determine Micronutrient Status***

Blood will be collected and stored using zinc free syringes and vials. Serum will be stored at -70° C until analysis of retinol and zinc. Serum zinc will be measured by atomic absorption spectrophotometry (ASS). Serum retinol and tocopherol will be measured using reverse phase high performance liquid chromatography (HPLC). Serum retinol binding protein (RBP) will be measured by ELISA. All of these determinations are currently being performed at the University of Natal School of Medicine.

***Determination of HIV status and CD4 count***

Samples will be tested for HIV using Organon Teknika quantitative HIV RNA assay. This assay has a sensitivity of 1600 copies per 50 μl sample. This is equivalent to 80 copies per ml.

After discussion with Professor Sharon Cassol, who directs the Africa Centre HIV Laboratory, we have modified the algorithm for determining HIV infection. It will now be as follows:

- *Samples collected at 6 and 18 weeks will be tested real time with Organon Teknika assay.*
- *If not detectable (<80 copies/ml), then the assessment is NEGATIVE ASSAY (NOT HIV-INFECTED)*
- *If 80-10,000 copies/ml, an additional sample will be obtained from the child >72 hours after the initial sample. If this second sample is >10,000 copies/ml the child will be classified as HIV-infected. If < 10,000 copies/ml the child will be classified as HIV-uninfected.*
- *If the initial sample is >10,000 copies/ml a second sample will be obtained. If there are >10,000 copies/ml in the second sample then the child will be classified as HIV-infected, and the parents or guardians informed accordingly. In the rare event that the second sample has a concentration < 10,000 copies/ml, a third sample will be obtained. Classification will then be based on the results of this third sample (< 10,000 copies/ml, HIV-uninfected; > 10,000 copies/ml, HIV-infected).*

This algorithm for diagnosis is consistent with that recommended by the Ghent International Working Group on Mother-To-Child Transmission of HIV.

We felt it necessary to add this second sample when ascertaining infected individuals to ensure that we give to parents the most accurate information possible on the status of their child. Children on whom a second sample cannot be obtained (when required) will be excluded from participation in the study.

CD4 cell counts will be measured on 1 ml of heparinized blood using a fluorescent activated cell sorter. Samples will be collected for HIV RNA three monthly from 6 month to 24 months of age, and for CD4 counts at 12, 18 and 24 months of age.

## Appendix VI Grading of Skin Reactions for nevirapine related reports

Although infants of mother who are HIV-infected will receive only one dose of NVP after delivery, we will continue to monitor for skin reactions related to nevirapine treatment. We will use the same grading systems as used in HIVNET O12, O23 and PACTG 316.

**GRADE 1: Erythema with or without pruritus.**

Management: manage pruritus and minor accompanying symptoms with antihistamines, antipyretics and/or non-steroidal anti-inflammatory medications.

**GRADE 2 A:** **Diffuse erythematous macular or maculopapular cutaneous eruption or dry desquamation with or without pruritus, but WITHOUT constitutional findings** (as described in grade 3); OR typical target lesions without blistering, vesicles, or ulcerations in the lesions.

Management: manage pruritus and minor accompanying symptoms with antihistamines, antipyretics and/or non-steroidal anti-inflammatory medications.

**GRADE 2 B: Urticaria**

Management: manage pruritus and minor accompanying symptoms with antihistamines, antipyretics and/or non-steroidal anti-inflammatory medications.

**GRADE 3: A. Diffuse erythematous macular or maculopapular cutaneous eruption or dry desquamation with or without pruritus but WITH any of the following constitutional findings considered related to study drug:**

1. 5 times upper limit of normal AST, ALT or twice baseline values if baseline was higher than upper limit of normal.

2. Fever, >39C

3. blistering and/or vesiculation of cutaneous eruptions

4. any site of mucosal lesions

OR

**B. Angioedema**

OR

**C. Exfoliative dermatitis defined as severe widespread erythema and dry scaling of skin with generalized superficial lymphadenopathy, AND with other constitutional findings such as fever, weight loss, hypoproteinemia possibly related to study drug**

OR

**D. Diffuse rash and serum sickness-like reactions defined as a clinical syndrome complex manifested as fever, lymphadenopathy, edema, myalgia, and/or arthralgia**

OR

**E. Diffuse cutaneous eruptions, usually starting on the face, trunk or back, often with prodromal symptoms PLUS one of the following:**

1. Cutaneous bullae, sometimes confluent with widespread sheet-like detachment of skin (<10% body surface area) (Nikolski's sign) (Stevens' Johnson Syndrome).

2. Two or more anatomically distinct sites of mucosal erosion or ulceration not due to another cause.

**Grade 4: Diffuse cutaneous eruptions**, usually starting on the face, trunk or back, often with prodromal symptoms PLUS cutaneous bullae, sometimes confluent with widespread sheet-like detachment of skin (>10% body surface area) (Nicholski's sign) (Stevens' Johnson Syndrome/Toxic Epidermal Necrolysis overlap syndrome).

Note:

- 1. A prodromal syndrome consisting of clinical symptom complex manifested as fever (>39C), malaise, cough, lymphadenopathy, edema, myalgia, and/or arthralgia may occur prior to the development of cutaneous manifestations of Stevens Johnson Syndrome (SJS), Toxic Epidermal Necrolysis (TEN) or the SJS/TEN overlap syndrome.
  2. All grade 3 or 4 skin rashes will be reported to the principal investigator within 48 hours of the event. An adverse event report is submitted within 48 hours.

## Appendix VII Forms

The data collection forms are being developed using special software (Teleform) and cannot be printed without this application. We can send the latest version of the forms if requested.

## Appendix VIII Multiple micronutrient supplementation

Multiple Micronutrient supplementation

|  |  |  | **%** | **mg per** |
| --- | --- | --- | --- | --- |
| **Ingredients** | **Claim** | **Product Form** | **Over RDA** | **Tablet** |
|  |  |  |  |  |
| Vitamin A | 375 mcg RE (1250 IU) | A Acetate 325 CWS/F | 35 | 5.2 |
| Vitamin D | 5 mcg | D 100 CWS | 35 | 2.7 |
| Vitamin E | 6 mg a-Toc. Equ. | E 50 CWS/F | 10 | 13.2 |
| Vitamin K | 10 mcg | 0.5% Trit | 50 | 3 |
| Vitamin C | 35 mg | Fine C Powder | 5 | 36.75 |
| Vitamin B1 | 0.5 mg | Rocoat 33 1/3% | 10 | 1.65 |
| Vitamin B2 | 0.5 mg | Rocoat 33 1/3% | 10 | 1.65 |
| Vitamin B6 | 0.5 mg | Rocoat 33 1/3% | 7.5 | 1.61 |
| Vitamin B12 | 0.9 mcg | 0.1% WS | 30 | 1.17 |
| Niacin | 6 mg | Niacinamide | 5 | 6.3 |
| Folate | 150 mcg | 10% Triturated | 25 | 1.88 |
| Fe | 10 mg | Ferrous Fumerate(32.87%Fe) |  | 30.42 |
| Zn | 10 mg | Zn Gluconate (14.35%Zn) |  | 69.68 |
| Cu | 0.6 mg | Cupric Gluconate 14% Cu |  | 4.3 |
| Iodine | 50 mcg | 1% Potas.Iodide Trit |  | 5 |
|  |  |  |  |  |
|  |  |  |  |  |
| Milk Solids |  |  |  | 1100 |
| DiPac |  |  |  | 600 |
| 6X Sugar |  |  |  | 600 |
| Fructose |  |  |  | 240 |
| Flavor |  | Pina colada |  | 52.4 |
| Citric Acid |  |  |  | 22.4 |
| Ca. Carbonate |  | Calcium carbonate 90 A |  | 53 |
| Mg Stearate |  |  |  | 13.5 |
|  |  |  |  |  |
|  |  | Tablet Wt. (mg) |  | 2865.81 |
